# Economic costs of global forest protection may be overstated

**DOI:** 10.1038/s41467-026-73569-0

**Published:** 2026-05-20

**Authors:** Prakash Nepal, Anthony Waldron, Jeffrey P. Prestemon, Trisha Gopalakrishna, Martin Jung

**Affiliations:** 1 USDA Forest Service, Forest Products Laboratory, Madison, WI USA; 2 Conservation Research Institute, Cambridge University, Cambridge, UK; 3 Working Ant Consultancy Cambridge, Cambridge, UK; 4 USDA Forest Service, Southern Research Station, Research Triangle Park, NC USA; 5 School of Biological Sciences, University of Bristol, Bristol, UK; 6 Environmental Change Institute, School of Geography and the Environment, University of Oxford, Oxford, UK; 7 International Institute for Applied Systems Analysis (IIASA), Laxenburg, Austria

**Keywords:** Environmental economics, Ecology, Social sciences

## Abstract

Protecting the world’s remaining forests is central to climate and biodiversity goals, but is often assumed to impose large opportunity costs on forest-land producers. These costs are typically estimated by summing local foregone production, without accounting for market feedback. Here, we applied partial-equilibrium models to three scenarios protecting an additional 471–863 million hectares of forest, assuming a global 30% protection target by 2030. Despite reductions in harvestable area and roundwood production, global net output value in the forestry sector increases modestly, driven by price increases under reduced supply. Most countries experience gains, although some incur losses. Despite broader economic impacts, including consumer welfare, substitution effects, and cross-sector response not being captured in this analysis, these results may indicate that approaches ignoring price adjustments may overestimate producer-side opportunity costs of large-scale forest protection.

## Abstract (web summary)

The study finds that large-scale forest protection under 30×30 could increase net forestry sector value as reduced supply raises prices, suggesting producer losses may be smaller than commonly assumed

## Introduction

Conservation is often perceived as economically costly, contributing to ongoing debates about environmental protection worldwide^1–3^. Forests present one of the most important examples of this problem. Protecting the world’s forests is key to halting biodiversity loss and slowing global warming, and indeed to supporting the livelihoods of about 1.3 billion often-marginalized forest-dependent people^4–7^. However, the global forestry (wood products) sector also employs more than 33 million people^8^ and contributes more than 1.5 trillion dollars per year to national economies^9,10^. Any initiative to protect forests, therefore, generates objections of “opportunity costs” – the economic loss suffered when producers are no longer allowed to exploit a forest^1–3^ – and the resultant political and industry opposition.

To overcome this perceived conflict between commercial production and climate/biodiversity needs, various studies have estimated the cost of conserving global forests, primarily through “buying out” or compensating the value of the foregone production^11–13^. However, the estimated costs are often so high – hundreds of billions of dollars per year, or several trillion dollars by 2050^11–13^ – that they represent a major barrier to action.

The calculation method behind these very high costs first estimates the value of potentially “lost” production for each individual hectare of forest, then simply sums up those values for every forest hectare protected worldwide. However, such an approach ignores two key economic principles, causing it to potentially exaggerate the true cost. First, a cut in production can sometimes benefit producers, by making their commodity scarcer and thus increasing the price they receive. If production goes down but price goes up, then the bottom line (“output value” in the jargon) can actually increase. This phenomenon is exemplified by OPEC’s (Organization of the Petroleum Exporting Countries) strategic imposition of production limits to enhance oil prices and profits for its members. Second, reduced production in one country is frequently offset by increased production elsewhere, leading to muted impacts on global consumption. Due to these two principles, the economic impacts of ambitious, large-scale forest protection may not be accurately captured by adding together thousands of small-scale opportunity costs. They are more appropriately evaluated using models that capture the interplay among price, demand, harvest volumes, trade (exports and imports), and revenues, in each country and in the world market overall.

The global commitment to protect 30% of all land for nature (also known as 30×30 or Target 3 of the Global Biodiversity Framework (GBF)) is one of the most ambitious nature conservation initiatives to date. With only 6.5% of forests currently under effective protection^14^, expanded forest protection could be central to 30×30’s implementation, implying that profits in the global wood- and forestry-product sector could be strongly impacted. Global forests under 30 × 30, therefore, provide an important test case of how current approaches to opportunity costs may be exaggerating the economic downside of large-scale conservation, and how full economic modelling could change our understanding of those costs. Here, we apply a partial equilibrium economic model to assess the impact of 30×30 on the global forestry sector’s bottom line. We find that, in contrast to the widely held expectation of an automatic loss, ambitious forest protection is projected to cause a modest increase in the forestry sector’s bottom line, although with a finer-scale pattern of winners and losers. We explain why this occurs and identify the countries most affected. Lastly, we outline the implications of our results for global environmental policy more generally.

We note here that a few recent studies have found similar outcomes, i.e., these costs may be far lower than previously assumed, and in some cases even net beneficial once broader economic feedbacks, avoided damages, and ecosystem-service benefits are considered. For example, Kedward and Poupard^15^ synthesize recent modelling exercises and report that the opportunity costs of implementing the 30×30 target are often modest, particularly when accounting for the value of ecosystem services, reduced climate-related damages, and the stabilizing effects of conservation on long-term economic systems. Similarly, Waldron et al.^16^, whose scenarios underpin the conservation pathways examined here, report positive economic effects from expanding protected areas. However, their estimates reflect gross output changes in the forestry sector and do not deduct harvest and transport costs, making them unsuitable for assessing forestry sector net output values. Similarly, following Waldron et al.’s^16^ approach, Johnson et al.^17^ estimated the cost of implementing 30×30 policies using an integrated ecosystem-economy model (GTAP-Invest) and found the total cost of such policies to be modest at $115 billion (0.1% of global GDP), and to be only $13 billion if the economic benefits of avoided CO_2_ emissions were accounted for.

The present study builds on this literature by providing a net output perspective that explicitly incorporates production costs within a sectorally detailed modelling framework that captures projected shifts in prices, production, consumption, and trade of roundwood because of increased forest protection. By focusing on net output value within the forestry sector, our analysis complements existing work and helps refine the understanding of how opportunity costs behave under large-scale forest protection scenarios.

## Results

### The opportunity costs of global forest conservation

**Fig. 1:**
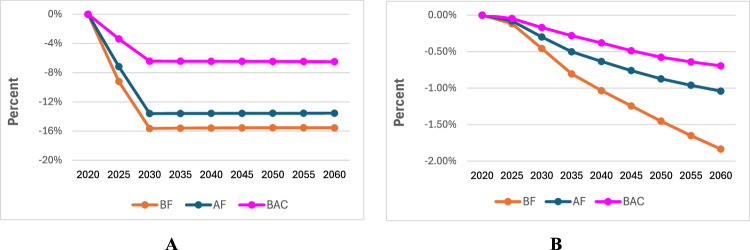
Projected impact of 30 × 30 on harvestable forest area and roundwood production relative to the reference. **A** Change in harvestable forest area (%). **B** Change in roundwood production (%). BF Biological-focus scenario, AF Agroeconomic-focus scenario, BAC Biological-agroeconomic compromise scenario.

**Fig. 2:**
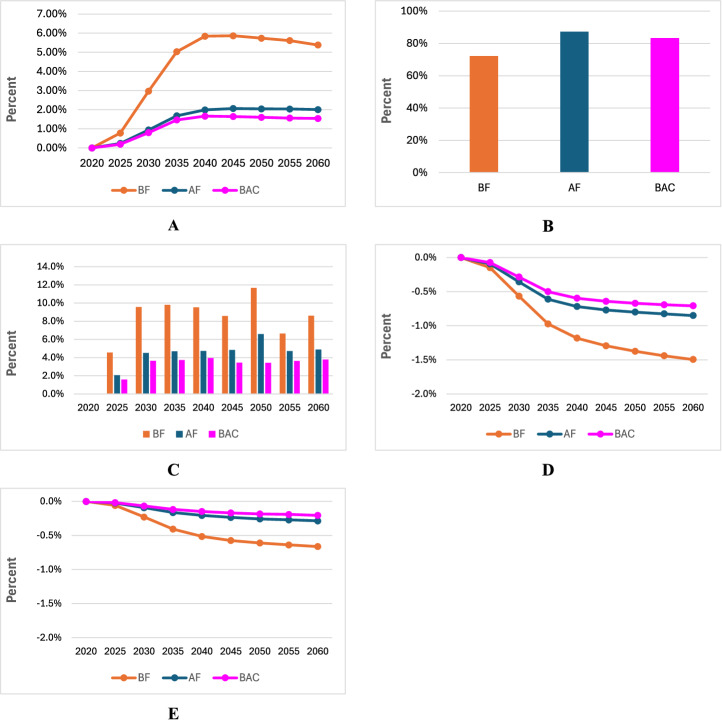
Projected impact of 30 × 30 on net output value, roundwood price, and wood demand (consumption) relative to the reference. **A** Change in net output value (NOV) due to implementation of 30 × 30 (%). **B** The proportion of countries that had a positive NOV change after 30 × 30. **C** Change in roundwood price (%). **D** Change in finished solidwood consumption (demand) (%). **E** Change in paper consumption (demand) (%). BF Biological-focus scenario, AF Agroeconomic-focus scenario, BAC Biological-agroeconomic compromise scenario.

In our results we found that, as expected, 30 × 30 reduced the total harvestable forest area by 6.4% – 15.6%, causing a reduction in roundwood production of 0.7–1.8% (depending on scenario, Fig. 1 and Supplementary Data 1). Despite these two decreases, annual global net output value (NOV) increased by 1.5% – 5.4% by 2060, representing a cumulative gain for 2025–2060 of $192 billion – $671 billion (Fig. 2, Supplementary Table 1). Interestingly, the BF (biological -focus) scenario generated the largest reduction in harvestable area but also the largest boost to NOV (Fig. 2).

This pattern of lower production but higher NOV suggests that, under 30×30, prices rise by more than production falls. The Global Forest Product Model (GFPM) results indeed show global roundwood prices rising between 4.5% and 9.6% (relative to the baseline scenario), driving up also the prices of finished wood and paper products by 0.3% to 3.0% depending on the scenario and product (Fig. 2, Supplementary Table 2). These price rises are possible because demand for wood products remains strong, even when the commodity is scarcer and more expensive (Fig. 2, Supplementary Table 3).

At national and regional levels, we found a more detailed pattern of winners and losers. At the national level, 87% of countries were projected to increase their NOV under AF (agro-economic focus scenario), 83% under BAC (biological-agroeconomic compromise scenario), and 72% under BF (Fig. 2, Supplementary Table 4). In BF, increases and decreases for individual countries were notably larger than in other scenarios, and therefore notably different from the global aggregate gain of 1.5–5.4%. For example, 70 countries in BF had projected increases of at least 10%, compared to four or fewer countries in other scenarios (Supplementary Table 1). Major beneficiaries included many developing countries such as Madagascar, Mali, Sierra Leone, Tanzania, and Botswana. However, twelve countries in BF also had particularly large losses (of >20% and up to 57%), including Malaysia, Rwanda, Malawi, and Australia (Supplementary Table 1). It is important to highlight that countries like Malaysia and Australia see large forestry losses because, after extensive deforestation, they have limited forested area left. Placing the last remaining forested areas off-limits to production, therefore, has an outsized economic impact. However, the last forest areas are also the last remaining habitat for those countries’ globally important biodiversity. Exploiting them could therefore cause multiple national (or global) extinctions.

**Fig. 3:**
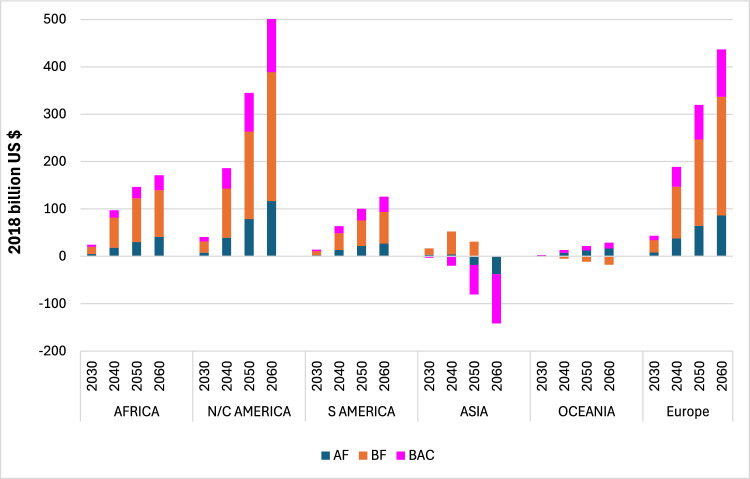
Regional differences in the projected impact of 30×30 on net output value, across three scenarios. BF Biological-focus scenario, AF Agroeconomic-focus scenario, BAC Biological-agroeconomic compromise scenario.

At the regional level, all regions but one experienced a positive NOV benefit, including an increase of 2.0–6.4% for Africa and 4.2–12.1%, for Europe (Fig. 3, Supplementary Table 1; Oceania, alone, had positive changes under AF and BAC but not under BF). The one regional exception was Asia, which had projected NOV losses under AF and BAC (−0.8% and −2.1%), but only just broke even under BF (+0.01%). Asia also had the highest projected proportion of loss-making countries of any region (30–40%), driven by multiple countries with projected losses in the Middle East and Central Asia. Projected results for all 180 countries evaluated are provided in Supplementary Data 2.

### Sensitivity tests and caveats

To test whether our conclusions were sensitive to the parameterization of their main modelled drivers (the price effect and the production cost estimates), we re-ran the analysis with various different parameterizations. First, we adjusted the price elasticity parameter in either direction by +/− 15% to test whether any plausible deviations from baseline elasticities would be sufficient to drive net NOV negative (in modelling, a higher price elasticity limits producers’ abilities to raise prices, making it harder for GFPM to project a positive NOV effect). This range provides a moderate but meaningful perturbation that allows us to identify how far the sign of NOV is robust to reasonable uncertainty in elasticity estimates. Second, we tried varying production costs by 4.5% and 9% in either direction (see Supplementary Table 5), noting that higher costs will decrease NOV gains. A 9% increase in total cost is designed to showcase an underlying 25% increase in timber transport costs, which is the main sectoral cost-type likely to be affected by greatly-expanded forest protection. We acknowledge that, in reality, the industry would act quickly to offset such a 25% cost increase, so the 9% sensitivity test is fairly extreme. Decreases in costs could occur if 30×30 constraints motivate innovations in tree growing, harvesting, or transport, or induce efficiency enhancements via supplier consolidation.

In the sensitivity test results, we found that unsurprisingly, reducing elasticity or costs generated larger NOV increases than in the main analysis (Supplementary Tables 6–8 and Supplementary Data 3–5). Of more potential interest are the results of the tests that made it harder to reach our conclusion of a positive NOV effect. For those tests, we found that increasing price elasticity by 15%, or production costs by 4.5%, did not alter the original conclusion, although the projected increases were smaller than before (Supplementary Tables 6 and 7 and Supplementary Data 3 and 4). In the most “profitable” scenario (BF), even the 9% increase in costs did not alter this conclusion: NOV still increased by 2.4% (Supplementary Table 7). In the other two scenarios, however (AF and BAC), 9% higher costs caused projected global NOV to decline by 0.9% and 1.4% respectively, equivalent to an average (mean) of $19-28 million per country per year, with losses concentrated in Canada, China, Russia, India, Turkey, and Thailand (Supplementary Data 4). Overall, the conclusion of a positive economic impact was therefore largely robust to multiple parameter changes. In the worst-case scenario, the overall opportunity cost would still be minimal. We caution that forestry-sector modelling in GFPM does not capture the possibility that, under persistently high wood prices, substitution may eventually occur toward alternative materials like plastics, concrete, or steel, weakening demand and price effects.

## Discussion

Although removing forest land from production is critical to achieving global climate and biodiversity goals, previous studies have suggested that this could cost up to half a trillion dollars per year at current prices, mostly due to the high opportunity costs^11,12^. An update of the Stern Review similarly projected protection costs of $1.38 billion per country per year (at 2025 values)^18^. However, for the global wood- and forest-product industry – the economic sector in the front line of forest-protection opportunity costs – our model projected not a high cost, but a small benefit. This occurs because, at a large scale, price effects mitigate reduced production, just as OPEC reduces oil production to increase profits. And as happens with oil, the wood/forestry sector’s bottom line was improved due to market effects, despite reductions in the amount of harvestable forest and lower production volumes. Even under the most conservative interpretation of our results, the opportunity costs of global forest protection would be minimal.

Our results also suggest a much broader issue with current opportunity cost methodologies. Simply summing up multiple, small-scale estimates of lost production, as current methods do, is likely to give reasonable results at smaller scales. But at large scales, where market effects will influence results, only full economic modelling can provide the necessary accuracy. Indeed, the small-scale approach can generate highly misleading exaggerations of the costs of large-scale initiatives. Two recent grey-literature studies that also used complex models have found non-intuitive outcomes similar to ours, for sectors outside forestry. Waldron et al.^16^ studied the impact of 30×30 on agricultural NOV, using several integrated assessment models that incorporate market and trade effects, and projected only a small NOV change, with losses and gains of ~1% both possible. Johnson et al.^17^ applied a partial equilibrium model to a different set of 30×30 scenarios and found that, in GDP terms, the global GDP opportunity cost could be as little as $13 billion per year, far below $300-400 billion calculated in earlier studies^19^.

If current methodologies are substantially overestimating the opportunity costs of global environmental action, the implications are far-reaching. Much of the friction that hinders the achievement of climate and biodiversity goals comes from the belief that large-scale nature protection could substantially reduce economic output. As a result, conservation economics has put substantial (and often expensive) effort into either compensating for those losses (opportunity costs) or justifying them. Alongside the half-trillion-dollar climate example above, global compensation strategies with potentially high financial costs also include carbon credits and emissions trading markets^20,21^, payments for ecosystem services^22^, direct international assistance, biodiversity offsets, and debt-for-nature swaps^23–25^. All these strategies could have excessively high cash expenditures if they use exaggerated opportunity cost estimates. Overpayments for opportunity costs are economically inefficient and may even be deemed unaffordable (in which case no pro-environment spending occurs). For countries that genuinely experience large opportunity costs, on the other hand, full economic models give a much better sense of the true magnitude of compensation needed. This would help avoid difficult political situations such as that in Palau, where the population has come to realize that ambitious conservation has lowered incomes without sufficient compensation, in contradiction to earlier assurances^26^.

An alternative strategy to compensation is avoiding opportunity costs in the first place. In the extreme, this includes blocking the implementation of environmental protections upfront. Such blocking is more likely to occur when opportunity costs are exaggerated. More widespread application of full economic models could therefore prevent governments from missing low-cost opportunities for environmental protection. More subtly, cost-avoidance can involve watering down the effectiveness of existing nature-conservation efforts. An example is the common practice of creating protected areas in remote areas to minimize opportunity costs, often causing a failure to spatially capture threatened biodiversity^27,28^. Interestingly, our results suggest that this approach does not always have the simple, positive economic impact that it aspires to. Our AF and BAC scenarios were designed to avoid protected-area opportunity costs in exactly this way, whereas BF was not. AF and BAC did succeed in reducing the largest single-country losses: for example, Rwanda’s loss shrank from 57% in BF to 3% in both AF and BAC, and Malaysia’s loss shrank from 36% in BF to 0.4% in BAC and indeed, became a 4% gain in AF (Supplementary Table 1). However, the two strategic cost-avoidance scenarios also reduced global NOV gains by over 50%. Indeed, only one country achieved a NOV gain of >10% in BAC and only four countries in AF, compared to 70 countries attaining that large benefit in BF (the non-cost-avoiding scenario, Supplementary Table 1). Pre-avoidance of opportunity costs could therefore substantially harm global market outcomes and reduce economic gains for many countries; this would be the trade-off for reducing losses in a smaller number of negatively-impacted countries.

The substantial differences we observe between global results and country‑level outcomes highlight the importance of distinguishing between regional analyses and global market‑integrated assessments when evaluating the opportunity costs of large‑scale conservation commitments such as 30 × 30. Regional studies provide essential detail on local forest conditions, management practices, and ecological constraints. For example, Regelmann et al.^29^ show that implementing the EU Biodiversity Strategy in Germany could reduce harvest volumes by 13–44% and lower forest‑owner revenues by 14–45%, driven in part by increased deadwood retention requirements that divert 15–19% of merchantable wood from the market. Such studies are indispensable for designing national implementation strategies and understanding local trade‑offs. However, regional models cannot capture the system‑wide market adjustments that occur when conservation policies are implemented across multiple countries. Reductions in timber supply in one region may raise global prices, shift production to other countries, or alter international trade flows, mechanisms that directly influence opportunity costs but lie outside the scope of national or sub‑national analyses. As a result, regional studies may overstate opportunity costs when interpreted in a global policy context, because they do not account for compensatory production or scarcity‑driven price effects elsewhere.

Our global modelling approach complements this literature by capturing these cross‑border dynamics and provides estimates of system‑wide producer opportunity costs under coordinated international conservation efforts. While global models necessarily abstract from local ecological and institutional detail, they offer critical insights into leakage, redistribution of production, and the distributional consequences of conservation across countries. Taken together, these perspectives underscore the value of a hybrid approach, one that integrates global market dynamics with region‑specific ecological and management constraints, to support equitable and effective policy design for achieving 30 × 30.

Our analysis finds that the magnitude and direction of NOV effects vary widely, reflecting differences in baseline production structures, trade orientation, processing capacity, and the relative importance of forest‑based industries. Countries with large, export‑oriented forest sectors tend to experience stronger NOV gains driven by scarcity‑induced price increases, whereas countries with smaller or more domestically oriented sectors may see modest gains or NOV losses. These patterns are consistent with broader evidence that the economic consequences of large‑scale forest protection are not uniform across regions. Such distributional heterogeneity across countries has important implications for the global implementation of the 30×30 target, indicating the need for policy designs that explicitly account for uneven impacts across countries. Nations that benefit less from scarcity‑driven price effects or that face higher adjustment costs may require targeted international support, including financial transfers, capacity‑building, or mechanisms to help them capture a greater share of value added in forest‑product markets. Conversely, countries that experience larger NOV gains may be better positioned to contribute to global conservation financing. Recognizing these distributional patterns is essential for developing equitable and politically feasible pathways for achieving 30 × 30. Future work should explore these cross‑country differences more deeply, including how they interact with broader welfare effects, substitution dynamics, and the institutional capacity required to implement large‑scale forest protection.

Although our study offers important insights into potential forest‑sector responses of expanded forest protection under 30×30, several inherent limitations remain, and therefore, findings should be interpreted with caution. This analysis relies on a partial market equilibrium modelling framework that focuses exclusively on the forestry sector. By design, the model holds conditions in other sectors constant and therefore does not capture cross‑sectoral shifts that may arise when conservation policies alter land availability or timber supply. Potential interactions with agriculture, energy, and other land‑using sectors, such as shifts in crop production, changes in biomass demand, or broader land‑use competition, therefore remain outside the scope of this analysis and warrant further investigation. In addition, partial market equilibrium approaches cannot represent macroeconomic feedbacks or financial‑sector channels such as shifts in land values, collateral dynamics, or credit conditions (e.g., see Kedward and Poupard^15^). The results reported here therefore, should be interpreted as sector‑specific effects rather than comprehensive economy‑wide outcomes.

Similarly, partial market equilibrium models such as GFPM cannot capture how reduced timber availability or higher wood prices might affect non-wood material-using industries (e.g., steel, concrete, engineered composites), nor do they show how feedbacks from those sectors might in turn influence wood product markets. The absence of explicit substitution dynamics has two important implications. First, if higher timber prices induce greater use of alternative materials, the resulting reduction in wood demand could moderate some of the price and revenue effects estimated here. Second, substitution may generate environmental spillovers that fall outside the scope of a forestry sector model. For example, replacing wood (a comparatively low-carbon construction material) with steel or concrete could increase life cycle emissions, thereby offsetting some of the climate benefits associated with forest conservation. Conversely, in applications where substitution is technologically or economically constrained (e.g., see Kedward and Poupard^15^), the effects estimated by the model may be closer to realized outcomes. Because these dynamics are not represented, the estimates reported here should be interpreted as upper-bound estimates under the assumption of limited substitutability across material classes. Incorporating substitution effects would require a multi-sectoral modelling framework, such as computable general equilibrium (CGE) models or integrated assessment models (IAMs). However, while CGE models can represent cross-sectoral interactions, such models typically sacrifice the detailed representation of forest product markets that a partial-market equilibrium model provides. The present analysis, therefore, prioritizes sectoral detail over economy-wide integration, and the findings should be interpreted within the boundaries of the forestry sector.

A further limitation of this analysis is that it does not capture the full set of societal or economic costs associated with expanding protected forest areas, such as avoided damage values or impacts on final consumers. We did not quantify these values here, because our interest was in the producer opportunity cost, which drives most of the perceived cost of protecting global forests, most of the powerful political opposition to expanding land conservation, and which (by definition) was the commercial value of the foregone wood products. Nevertheless, we note that there are possible all-of-society impacts from forest protection that a forestry model cannot capture on its own. On the negative side, the benefit to producers of receiving higher prices implies a disbenefit to the consumers paying those prices, although the distribution of that change across potentially billions of wood-product consumers has very complex equity and economic implications, lying beyond the scope of the model here. On the positive side, a comprehensive social cost–benefit assessment would also require valuing the economic and societal losses avoided thanks to forest protection, such as biodiversity losses, diminished water regulation, flood mitigation, soil retention, carbon storage, and other regulating and cultural services. Existing evidence emphasizes the magnitude of these benefits: for example, Kappen et al.^30^ estimate that the non‑commercial social, environmental, and climate‑regulatory value of global forests constitutes more than 90% of total forest value, with lower and upper bounds of 50–150 trillion dollars. Such avoided‑damage values would generally reduce net social costs of forest conservation and may even yield net economic benefits, in ways that further interact with the producer and consumer effects associated directly with wood products.

In summary, changing the approach to opportunity costs, so that economic effects (such as the price effect) are more fully captured, would have multiple benefits. Concerns about the negative commercial impact of globally important conservation initiatives might be widely attenuated, removing barriers to action. Greater use of such models could uncover other global environmental policy propositions that have lower-than-expected adverse effects. And win-win outcomes that are hard to intuit, such as increases in output values driven by price increases under reduced supply, could be identified and acted upon, to capture both economic and environmental benefits for the future.

## Methods

### Protected forest area scenarios

Most decisions about 30×30 implementation have not yet been made, so to model its economic effects, we used three different scenarios of where new protected areas might be implemented. The three scenarios were taken directly from Waldron et al.^16^ and reflect a logic that when creating new protected areas, governments are likely to trade off biodiversity importance against economic or food-security considerations. The first scenario (called BF) is purely “biological -focused”, using Integer Linear Programming (ILP) to identify an optimal set of new protected areas without regard for economic consequences (see Waldron et al.^16^ for details). The second scenario (AF or “ agro-economic focus”) is “agricultural production focused”. It starts by identifying all current natural areas (including forests) that could require conversion to agriculture to efficiently meet future food-production needs (up to 2050) and removes them from possible protection, before running the ILP optimization as before. The third scenario (BAC, for “biological-agroeconomic compromise”) is a biodiversity/production compromise, i.e. a land-use planning compromise between biodiversity needs and future agricultural production needs (see Supplementary Methods 1 and Waldron et al.^16^). We note that future forestry production is threatened by agricultural expansion as well as by protection. By prioritizing agricultural production and biodiversity conservation simultaneously in our scenarios, we capture some of the complex interplay between these three competing land demands. Further details regarding the scenarios can be found in the Supplementary Methods 1.

### Projecting the impact of 30 × 30 on the global forestry industry

To model how 30×30 affects output values across the global forestry sector, we used the Global Forest Products Model (GFPM)^31–33^, a partial market equilibrium model that has been successfully applied to evaluate a range of questions about worldwide, regional, and country-level forest sector outcomes^16,31,34–36^. The GFPM models market equilibrium production, consumption, price, imports, exports, and revenues over time, for 14 categories of forest products in 180 countries and territories, solved by the model by maximizing the value of the products purchased by consumers at market equilibrium prices, minus their costs of production and transport (see Supplementary Methods 2 and Supplementary Fig. 1). It also projects changes in standing forest levels and wood volume levels, as trees grow naturally, are planted, or are removed in response to demand and price. Hence, it addresses the many, complex economic interplays that the simplified approach to opportunity cost ignores, including the possible increase in price and the shifting balances of production, consumption, and trade. The GFPM takes as its initial input the reduction (“shock”) in harvestable forest area implied by 30 × 30. It then projects the changes in production of roundwood and its downstream uses, and the evolution of prices, consumption, production, imports, exports, forested area, and final revenues, from 2025 to 2060. For comparability with other opportunity cost studies, we assume that protection fully prevents exploitation, acknowledging that this is not always the case on the ground^37^. Additional details of the GFPM model and scenarios input to GFPM are provided in the Supplementary Methods 2, 3.

The GFPM generates a gross output value (GOV) for the forestry sector (total forestry revenues per country). However, the more meaningful bottom-line metric is the net output value (NOV), which subtracts the costs of production (on which there is limited information) from the revenues earned (see Supplementary Methods 4). To convert GOV to NOV, we researched records of national forestry-production costs, collating results across 68 studies (see ‘Forestry cost estimates’ section below). The literature does not provide costs for several countries, so we took regional cost means and then applied sensitivity tests to our results (Supplementary Fig. 2, Supplementary Table 5). The tests also explore the possibility that costs themselves might be affected by 30 × 30 (see the ‘Sensitivity tests’ section below). Finally, we calculated the 30 × 30 impact as the difference between the NOV for a reference baseline (in which no new protected areas are created) and the NOV projections for each of the three scenarios. This differencing approach isolates the effects of protected-area expansion on market outcomes under each 30×30 scenario.

### Forestry cost estimates

Information on forestry costs in all 180 countries modelled by the GFPM is limited. To generate the most comprehensive database possible, we searched the global peer-reviewed and grey literature for estimates of forestry costs. We found and collated 68 country-specific studies from various publications and the citations and data therein (including previous collation studies)^38–43^. Many of those studies reported harvest costs only. However, post-roadside costs (transport to mill/port) are known to represent 25-50% of the total costs, making them potentially at least as large as harvest costs^44^. Transport costs are only sometimes paid by the logging company^44^, but we nevertheless chose to include them, in order to minimize the possibility of overestimating net financial outcomes (noting that the larger the cost estimate, the smaller and more potentially negative the 30 × 30 NOV impact becomes). Several studies on harvest costs included neither these large transport costs, nor the much smaller costs related to pre-harvest work. To overcome these lacunae, we used studies where either transport cost or pre-harvest cost (or both) was reported alongside harvest costs, to calculate the ratio between the harvest cost and the transport or pre-harvest cost. We then obtained the means of those ratios and applied them to impute missing pre- or post-harvest costs. We adjusted all the dollar values for inflation to 2018 constant-dollar values. The final estimated average regional total harvest costs (including harvest, pre-harvest, and transport to mill) are shown in column 4 of the Supplementary Table 5 and Supplementary Fig. 2. These were subtracted from the GOV model outputs to generate NOVs.

### Sensitivity tests

Transport costs are the costs that are most immediately likely to change from their current (database) values under 30 × 30, for example, if expanded forest protection causes spatial changes in logging sites and roads, and therefore in distances travelled. The large contribution of transport costs to total costs also makes such an effect potentially influential on the final conclusions about NOV. In practice, any increase in those costs will make it harder for the model to project an increase in NOV: if 30 × 30 increases GOV but increases costs more, then NOV will still be negative. To test the sensitivity of our conclusions to these possible cost differences, we recalculated NOVs after increasing transport costs by 12.5%, equivalent to a 4.5% overall cost increase. We also explored the impact of 25% higher transport costs, equivalent to a 9% overall cost increase, although this may be seen as extreme, not least because most wood-sector industries faced with such a large increase in one of their major costs would likely compensate for it urgently by cost-cutting, by raising prices, or by adjusting other operational aspects. For balance, we additionally tested the impact of cost decreases of 4.5% and 9%. A decrease in future costs may occur if technological advances lower input costs (per unit output), and indeed if 30×30 itself motivates greater efficiency.

Any increases in forestry earnings due to reduced supply are also strongly driven by the degree to which prices rise as production is cut. We therefore also sensitivity-tested the outcomes of modelling runs in which (i) supply was made more price-sensitive, implemented by multiplying the price elasticity of supply by 1.15; and (ii) supply was made less price-sensitive, implemented by multiplying the elasticity by 0.85.

The main text describes the results of these sensitivity tests, which generally confirmed the robustness of the overall conclusion that ambitious increases in forest protection under 30 × 30 leads to a slight increase in forestry and wood sector NOVs.

### Reporting summary

Further information on research design is available in the Nature Portfolio Reporting Summary linked to this article.

## Supplementary information

Supplementary Information

Description of Additional Supplementary Files

Supplementary Data 1

Supplementary Data 2

Supplementary Data 3

Supplementary Data 4

Supplementary Data 5

Reporting Summary

Transparent Peer Review file

## Data Availability

The authors declare that the data supporting the findings of this study are available within the paper and its supplementary information files. The latest version of the GFPM model is available at https://buongiorno.russell.wisc.edu/gfpm/. Data used to develop and calibrate the GFPM were compiled by the model developers from multiple published sources, which can be found in the user manual document, also available at the same weblink above. Two additional datasets were assembled by the authors: (1) estimates of protected forest area under the 30 × 30 target, derived from a spatial optimization procedure that integrated outputs from Integrated Assessment Models as described in the main text and the supplementary information, and (2) country-level estimates of timber harvest and transport costs, compiled from various published sources and detailed in the main text and supplementary information.
