# Supplementary material for: Economic costs of global forest protection may be overstated
Source: Nat Commun. 2026 May 20;17:6649. doi: 10.1038/s41467-026-73569-0 (PMC13381589; doi:10.1038/s41467-026-73569-0)
Supplement: Supplementary file 1 — Supplementary Information [file 41467_2026_73569_MOESM1_ESM.pdf]

Supplementary Information for

Economic costs of global forest protection may be overstated

Prakash Nepal[1]†*, Anthony Waldron[2,3]†, Jeffrey P. Prestemon[4], Trisha Gopalakrishna[5,6], Martin Jung[7]

†These authors contributed equally to this work

*Corresponding author. Email: Prakash.Nepal@usda.gov

[1] USDA Forest Service, Forest Products Laboratory; Madison, WI 53726, USA.

[2] Conservation Research Institute, Cambridge University, UK

[3] Working Ant Consultancy Cambridge; 2 Copley Hill Business Park, Cambridge CB22 3GN, UK

[4] USDA Forest Service, Southern Research Station; Research Triangle Park NC 27709, USA

[5] School of Biological Sciences, University of Bristol, Bristol BS8 1TQ, UK

[6] Environmental Change Institute, School of Geography and the Environment, University of Oxford; Oxford OX1 3QY, UK

[7] International Institute for Applied Systems Analysis (IIASA), A-2361, Laxenburg, Austria

Contents

Supplementary Methods 3
Supplementary Methods 1: Protected forest area scenarios 3
Supplementary Methods 2: The Global Forest Products Model 4
Supplementary Methods 3: Scenario input into GFPM 7
Supplementary Methods 4: Estimating changes in economic output due to 30x30 8
Supplementary Figures 12
Supplementary Figure 1. Flow of raw materials, intermediate products, and end products modeled in the GFPM. 12
Supplementary Figure 2. Estimated mean real cost of harvesting, extracting, and delivering roundwood to mills in various regions (2018 US dollars/m^3). 13
Supplementary Tables 14
Supplementary Table 1. Projected changes in Net Output Values (NOVs) in 30x30 scenarios relative to reference scenario. 14
Supplementary Table 2. Projected changes in world prices (%) of roundwood and manufactured wood products in 30x30 scenarios in 2030, relative to projected 2030 reference levels. 18
Supplementary Table 3. Percent changes in cumulative consumption and production of roundwood, finished solidwood products, and paper products in 30x30 scenarios relative to reference, 2025-2060. 19
Supplementary Table 4. Number and proportion of countries with positive changes in cumulative Net Output Values in 30x30 scenarios, 2025-2060. 20
Supplementary Table 5. Estimated mean harvest costs and assumed increases in those costs under two sensitivity tests. 21
Supplementary Table 6. Effects of a higher price elasticity on Net Output Values (billion 2018 US $, cumulative, 2025-2060) in world regions in 30x30 scenarios relative to reference scenario. 22
Supplementary Table 7. Estimated changes in Net Output Values (billion 2018 US $, cumulative, 2025-2060) in world regions in 30x30 scenarios relative to reference scenario, using base price elasticity. 23
Supplementary Table 8. Effects of a lower price elasticity of supply and higher and lower harvest costs on estimated Net Output Values (NOV) in major world regions in 30x30 scenarios relative to reference scenario. 24
Supplementary References 25

Supplementary Methods

Supplementary Methods 1: Protected forest area scenarios

To represent possible implementations of 30x30 and calculate their impact, we took three scenarios for the land that might be protected to achieve a global 30% target, and one reference scenario where there was no protection added beyond the current protected-area coverage. The scenarios were taken from The Waldron Report[1] but with a change of nomenclature: we used the scenarios REF, HPR, BPC and SSE from the Waldron Report but here, they are instead referred to as REF, AF, BAC and BF (in the same order).

The three scenarios envisage different levels of trade-off between economic and biodiversity priorities. The first, biological-focused 30x30 scenario ("BF"), applies an integer linear programming (ILP) process that allocates new land for protection by maximizing biological conservation (i.e., the number of species that would become Least Concern on the IUCN rankings of extinction risks), without regard for economic consequences. The biodiversity data for optimizing conservation priorities come from Jung et al. study[2]. The second, agro-economic-focused scenario ("AF") starts by removing from possible protection the natural land that an integrated assessment model (AIM) projects will be needed for future agricultural production (up to 2050), then seeks to maximize biodiversity outcomes on the remaining natural lands via the same ILP process. The third, biological-agroeconomic compromise scenario ("BAC"), represents a compromise between the first two 30x30 scenarios. It applies the BF ILP methods to expand protected areas from their current extent (about 17%, www. protectedplanet.net) up to 20%, without regard for economic consequences. The remaining 10% needed to achieve 30% coverage is then derived from all remaining land using the ILP process, except that in that remaining land, any areas needed for future agricultural production are masked out before the ILP process is run.

Land use data from the various IAM runs used in these spatial optimization procedures were obtained from the supplementary material[3] for the Leclère et al. study [4]. The reference scenario (REF) used the November 2020 protected area spatial layer from the World Database on Protected Areas (www.protectedplanet.net), kindly cleaned by the World Conservation Monitoring Centre for A.W. Half the expansion in protected area needed for 30x30 was modelled as being implemented in 2025, with the remaining half then implemented by linear interpolation up to 2030.

Supplementary Methods 2: The Global Forest Products Model

The Global Forest Products Model (GFPM) is a partial market equilibrium model of the global forest sector[5,6]. As a policy tool, GFPM has been widely applied to evaluate a range of questions relevant to worldwide, regional, and country level forest sectors[1,5,7–9]. A detailed description of the model's structure and its mathematical formulations are found in several publications (e.g.,[5,6,10]). Here, we briefly describe this model and our approach in modeling the effects of forest area changes on global forests and the forest products sector.

GFPM models markets for 14 categories of forest products (Figure S1) in 180 countries and territories including 50 in Africa, 47 in Asia, 37 in Europe, 22 in North America, 13 in South America and 11 in Oceania. The 14 products represented in the model include five raw materials (industrial roundwood, roundwood, fuelwood, recovered paper, and other fiber pulp), two intermediate products (mechanical and chemical pulp), and nine end products (sawnwood, plywood, particleboard, fiberboard, newsprint, printing & writing paper, other paper, fuelwood, and other industrial roundwood). For each year, the production, consumption, trade, and prices of roundwood and finished wood products are endogenously determined in GFPM by modeling

demand for end products and supply of roundwood feedstock[5,6]. In each year of a projection, the demands for end products are functions of exogenously projected GDP per capita and their endogenously projected prices. The forest stock of a country evolves as last year's forest stock plus the current year's growth minus the current year's harvest. The supply of roundwood is then specified as a function of the forest stocks in available forests and their endogenously projected prices. Forest stock growth (net of mortality) before harvest is modeled as a negative nonlinear function of forest stock density (i.e., forest stock per unit of forest area)[11], i.e., the rate of forest stock growth increases with declining stock density and decreases with increasing stock density. Consumption of raw materials (e.g., industrial roundwood) is derived from the demand for end products through the input–output coefficients (ratio of the quantity of industrial roundwood input used in manufacturing a product to the quantity of that product) and manufacturing costs (labor, capital, energy). Differences in input costs, input–output efficiency, manufacturing costs, transportation costs, and profits determine the comparative advantages of countries in producing and shipping a product[5,6]. For this analysis, we used the 2021 version of the GFPM, the latest version available at the time of analysis, which had a base year of 2017. Model projections were generated at five-year intervals, beginning in 2020 and extending through 2060.

GFPM is designed primarily as a policy analysis tool, facilitating an understanding of how forest products production, consumption, imports, exports, prices, and welfare are likely to change under a given or a combination of scenarios of economic changes (e.g., changes GDP), biophysical changes (e.g., changes in forest area, growth and inventory), changes in technology (e.g., changes in production capacity), and changes in trade and related policies (e.g., tariff and non-tariff related trade barriers)[7,10]. Such a capability in GFPM is enabled through the integration

of the classical four major components of forest sector models, including (i) timber supply (production of raw materials), (ii) processing industries (manufacturing of materials into products), (iii) demand for end products, and (iv) international trade. The market equilibrium quantities of production, consumption, trade and prices solved by the model for given year and a country is obtained by maximizing the quasi-net welfare of the world forest sector: the value of the products to consumers, minus their cost of production and transport[12,13] (Supplementary Equation 1).

$$\max Z = \sum_i \sum_k \int_0^{D_{ik}} P_{ik}(D_{ik}) dD_{ik} - \sum_i \sum_k \int_0^{S_{ik}} P_{ik}(S_{ik}) dS_{ik} - \sum_i \sum_k \int_0^{Y_{ik}} m_{ik}(Y_{ik}) dY_{ik} - \sum_i \sum_j \sum_k c_{ijk} T_{ijk} \quad (1)$$

where: *i* and *j* refer to countries and *k* refers to a product. *P* is price in U.S. dollars of constant value, *D* is final product demand, *S* is raw material supply, *Y* is quantity manufactured, *m* is cost of manufacture (labor, capital, and materials excluding wood and fiber), *T* is quantity transported, and *c* is freight cost (cost of transport plus tariff).

Thus, the GFPM is built on the general principle that the allocation of scarce resources in the short run is optimized by global markets, while the long run resource allocation is partly determined by the combination of market forces (e.g., projected prices) and policy changes affecting the forest sector. Policy changes may include laws or government programs affecting wood supply, regulations or incentives directed towards wastepaper recovery (i.e., the paper recycling rate), or alterations in tariff and non-tariff barriers that affect international trade.

Supplementary Methods 3: Scenario input into GFPM

For each scenario, we estimated the amount of forest that remained outside of the 30% protected area (the "harvestable forest area") in 2025-2030 (Supplementary Table S9), using the combined forest classes from the most recent year available (2015) of the high-resolution Global Land Cover (GLC) land use classification[14]. Note that this approach assumes that forest in protected areas is fully protected and is not available for harvesting activities. We acknowledge that some timber exploitation can currently occur in protected areas[15] but assume, for simplicity, that this becomes negligible after the implementation of 30x30.

GFPM uses and is calibrated with forest area reported by the Global Forest Resource Assessment[16]. Cross-checking showed that the FAO estimate of total forest area values for 2020 was different from the estimate derived using the GLC, which likely occurred because the FAO statistics are self-reported by countries whereas GLC land use classification data is satellite-based[14]. To align the GLC-based forest areas with the forest-area parameters taken as input by the GFPM, we used the percentage changes in GLC-based available forest area (per scenario and country) to generate a shock of the same percentage magnitude to the roundwood supply in GFPM. For a few countries, the projected percent changes in exploitable forest areas (particularly under the BF scenario) were so large that no forest stock would be available to harvest and manufacture wood products. While this would be a non-issue for modeling the scenarios for countries with little or no forest area or manufacturing activities, for seven countries in the BF scenario (only), such changes would prevent GFPM from identifying a market-clearing solution. To solve this problem, and for the BF scenario only, we adjusted

downward the unavailable forest area percentage by half for those seven countries: New Zealand, South Africa, Thailand, Czechia, Chile, Liberia, and Equatorial Guinea.

The harvestable forest area, in each year from 2025 to 2030 and in each scenario, was then input into GFPM. Specifically, the exogenously applied shocks (reductions in available forest area) were translated endogenously in GFPM into proportionate changes in available forest growing stocks (quantity of standing timber, in cubic meters), which the model then translates into change in the roundwood supply. In this analysis, 'roundwood' includes industrial roundwood, other roundwood, and fuelwood, which combined represent the primary wood products sector.

Supplementary Methods 4: Estimating changes in economic output due to 30x30

The projected changes in forest growing stock generate shifts in timber supply within the GFPM, with reductions in stocks causing backwards shift in the supply of roundwood for each country, leading to new market equilibria that characterize the forest sector outcomes of the 30x30 initiative. The model projection of the impact of each individual scenario on forestry economic outputs is initially based on the changes in the production, value and trade of roundwood, with effects on "downstream' products from roundwood worked out subsequently. Domestic roundwood prices are represented by the world price for a net exporting country, whereas for the net importing country, domestic prices are represented by world price plus freight cost (the cost of shipping products overseas)[5,6].

The economic consequences of 30x30 for the forestry sector were derived by comparing the equilibrium model solutions for the three 30x30 scenarios with the solutions for the reference scenario in terms of price, production, consumption, trade, and revenues. Background (non-

market-driven) changes in forest areas after 2030 were also endogenously projected by GFPM based on an environmental Kuznets curve[17], in which forest area change is negative at low GDP per capita, becomes positive and increases at higher GDP per capita, and then decreases and approaches zero at very high GDP per capita[11]. The main equilibrium result of interest is the gross output value (GOV), calculated for each scenario from the projected prices and quantities of roundwood production in each country (i.e. quantity * price). We projected these outcomes from 2025 to 2060 in five-year increments. Note that GDP per capita and all other economic variables remained the same in both the reference and the alternative scenarios, except for the available forest area, which allowed us to attribute the model outcomes to changes in available forests due to the three scenarios for implementation of the 30x30 initiative.

Finally, the net output value (NOV) of roundwood production was calculated by subtracting the average cost of harvesting and transporting roundwood from the projected GOV. The effects on net revenues of downstream manufacturing products production were not calculated, because that would represent a form of double-counting, since roundwood is the input to those manufactured wood products (see[18,19]).

We quantified the economic effects of 30x30 on forest product producers, finding that constrained supply would cause them to be paid more per unit and thus, counterbalance (or even overcompensate) any volume losses from increased land conservation. An increase in forest product prices implies negative effects on forest product consumers, in further counterbalance to the zero-sum or positive effects found for forest product producers. Although these welfare effects are important, our modelling focused on the opportunity costs to producers, for both a

conceptual and practical reason. Conceptually, the "cost" of forest protection, especially in the arena of climate change policy, refers specifically to the opportunity cost to producers and not to broader welfare. Since the main drive of the article is that those costs have been exaggerated, we preserve the same sense of cost as "producer opportunity cost". Moreover, the politically meaningful opposition to land conservation will typically come from producers fearing an opportunity cost, and not from consumers. On the pragmatic side, GFPM is also specifically designed to estimate producer effects rather than society-wide welfare effects.

We therefore emphasize that our analysis focuses on changes in NOV for roundwood producers, not the changes in producer and consumer economic surplus across the entire wood products sector. GFPM and comparable forest-sector models do not represent consumer or producer behavior for all products using explicit demand and supply equations, preventing estimates of total welfare. In GFPM, roundwood consumption is derived from the consumption of finished products (sawnwood, panels, and paper) and intermediate products (wood pulp) using input–output coefficients, rather than from a demand curve with an associated elasticity, preventing estimates of roundwood consumers surplus. Likewise, production of finished wood products is not determined by a supply curve but is derived from input–output relationships, which prevents estimation of producer surplus for downstream industries. As a result, the broader effects on roundwood consumers, downstream producers and consumers of finished products, and overall social welfare remain unknown and warrant future investigation using modelling frameworks that include explicit demand and supply functions for all types of timber and finished wood products.

Supplementary Figures

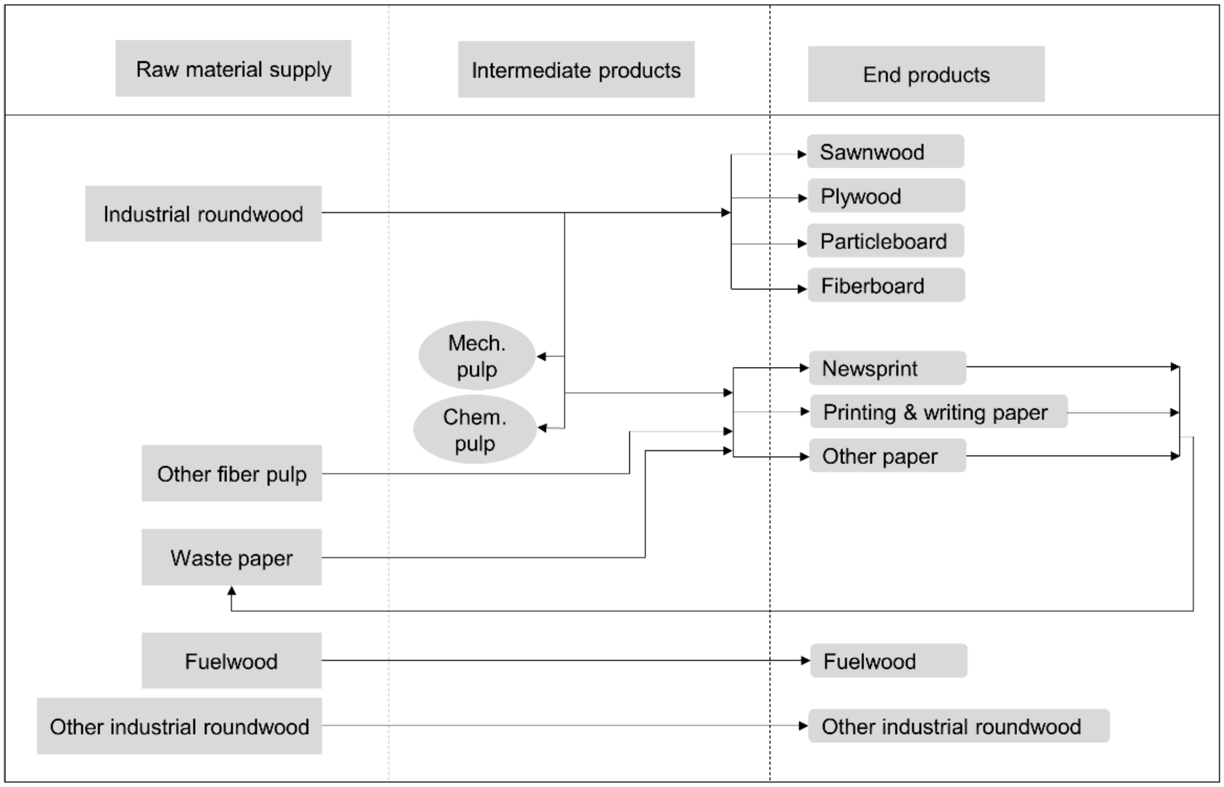

Supplementary Figure 1. Flow of raw materials, intermediate products, and end products modeled in the GFPM.

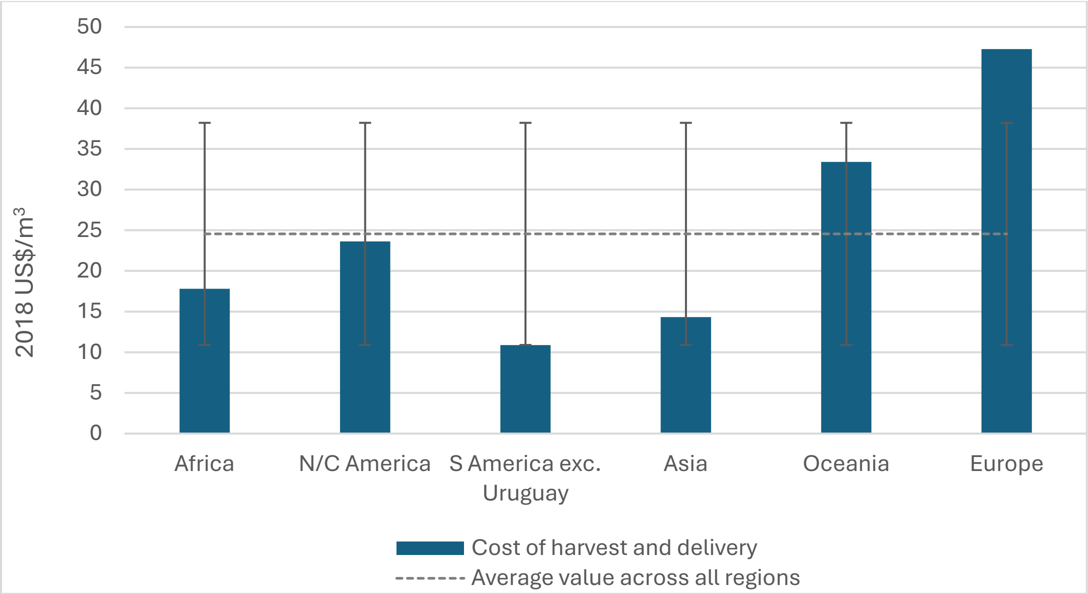

Supplementary Figure 2. Estimated mean real cost of harvesting, extracting, and delivering roundwood to mills in various regions (2018 US dollars/m^3). Error bars represent one standard deviation from the data mean.

Supplementary Tables

Supplementary Table 1. Projected changes in Net Output Values (NOVs) in 30x30 scenarios relative to reference scenario. Value columns represent absolute changes (cumulative million 2018 US $, 2025-2060) compared to REF, and % columns represent % change in cumulative NOVs compared to REF).

Region/Country	BF[1]		AF[2]		BAC[3]	
	Value	%	Value	%	Value	%
**AFRICA**	**98,552**	**6.4%**	**41,233**	**2.7%**	**31,371**	**2.0%**
Algeria	605	3.5%	423	2.4%	398	2.3%
Angola	1,419	10.9%	311	2.4%	453	3.5%
Benin	1,574	11.4%	481	3.5%	401	2.9%
Botswana	179	10.8%	39	2.3%	46	2.8%
Burkina Faso	3,466	9.1%	1,102	2.9%	909	2.4%
Burundi	1,275	10.0%	403	3.2%	331	2.6%
Cameroon	-763	-2.2%	162	0.5%	-1,404	-4.0%
Cape Verde	35	10.3%	10	3.0%	8	2.3%
Central African Republic	821	12.3%	-39	-0.6%	213	3.2%
Chad	1,898	11.1%	424	2.5%	456	2.7%
Congo, Republic of	-87	-0.8%	742	6.9%	27	0.3%
Côte d'Ivoire	2,215	8.9%	843	3.4%	673	2.7%
Djibouti	82	11.3%	22	3.0%	19	2.7%
Egypt	1,225	9.3%	324	2.5%	285	2.2%
Equatorial Guinea	-202	-4.4%	340	7.4%	257	5.6%
Ethiopia	23,023	10.7%	6,599	3.1%	5,704	2.7%
Gabon	-350	-4.1%	454	5.3%	175	2.1%
Gambia	84	2.8%	-18	-0.6%	-39	-1.3%
Ghana	10,557	11.2%	3,295	3.5%	2,780	2.9%
Guinea	2,769	11.1%	838	3.3%	705	2.8%
Guinea-Bissau	586	10.1%	191	3.3%	160	2.8%
Kenya	4,290	7.9%	1,661	3.0%	1,323	2.4%
Lesotho	330	6.5%	-21	-0.4%	-68	-1.4%
Liberia	1,637	8.9%	431	2.3%	45	0.2%
Libyan Arab Jamahiriya	-534	-21.7%	59	2.4%	83	3.4%
Madagascar	2,599	9.5%	798	2.9%	625	2.3%
Malawi	-5,311	-31.7%	456	2.7%	267	1.6%
Mali	1,526	10.5%	385	2.6%	401	2.7%
Mauritania	468	11.3%	131	3.2%	114	2.7%
Mauritius	0	-2.4%	0	2.8%	0	-0.6%
Morocco	-88	-0.5%	324	1.8%	255	1.4%
Mozambique	3,686	8.6%	402	0.9%	584	1.4%
Niger	2,664	11.3%	292	1.2%	629	2.7%
Nigeria	13,443	8.0%	3,515	2.1%	2,507	1.5%
Réunion	13	6.6%	6	3.3%	5	2.8%
Rwanda	-6,792	-56.5%	-321	-2.7%	-387	-3.2%
Sao Tome and Principe	1	0.5%	12	3.7%	9	3.0%
Senegal	1,152	8.6%	413	3.1%	306	2.3%
Sierra Leone	1,242	10.7%	378	3.3%	323	2.8%
Somalia	2,543	8.9%	364	1.3%	719	2.5%
South Africa	-4,609	-5.9%	996	1.3%	-1,086	-1.4%
Sudan	2,430	6.6%	878	2.4%	805	2.2%

Swaziland	-2,736	-53.2%	134	2.6%	79	1.5%
Tanzania, United Rep of	5,432	10.3%	1,854	3.5%	1,521	2.9%
Togo	807	8.9%	243	2.7%	195	2.2%
Tunisia	218	2.4%	236	2.6%	184	2.0%
Uganda	-3,005	-3.4%	2,946	3.3%	2,466	2.8%
Congo, Dem Republic of	19,396	10.9%	5,338	3.0%	4,925	2.8%
Zambia	5,312	10.9%	1,700	3.5%	1,422	2.9%
Zimbabwe	2,028	9.4%	676	3.1%	565	2.6%
**N/C AMERICA**	**271,972**	**10.9%**	**116,648**	**4.7%**	**120,829**	**4.8%**
Bahamas	5	9.4%	2	3.9%	2	2.8%
Barbados	-16	-42.6%	2	5.6%	2	4.3%
Belize	-1	-0.2%	16	4.8%	13	3.8%
Canada	82,899	11.0%	605	0.1%	32,932	4.4%
Saint Lucia	2	12.4%	1	3.7%	0	2.2%
Costa Rica	316	3.0%	601	5.8%	464	4.5%
Cuba	100	2.3%	233	5.4%	180	4.2%
Dominica	1	10.2%	1	4.1%	0	0.8%
Dominican Republic	138	7.7%	71	4.0%	60	3.4%
El Salvador	-141	-1.8%	193	2.5%	152	1.9%
Guatemala	4,023	11.4%	1,330	3.8%	1,132	3.2%
Haiti	343	9.6%	109	3.0%	81	2.3%
Honduras	1,342	8.9%	622	4.1%	521	3.4%
Jamaica	244	13.0%	118	6.3%	89	4.8%
Martinique	2	12.1%	1	3.3%	0	1.2%
Mexico	10,384	10.8%	4,713	4.9%	3,568	3.7%
Netherlands Antilles	0	-11.4%	0	3.5%	0	-19.2%
Nicaragua	792	7.7%	178	1.7%	129	1.3%
Panama	-244	-9.3%	-4	-0.1%	-50	-1.9%
Saint Vincent/Grenadines	1	8.6%	1	3.8%	0	2.1%
Trinidad and Tobago	-148	-35.8%	20	4.8%	15	3.7%
United States of America	171,926	11.0%	107,837	6.9%	81,539	5.2%
**S AMERICA**	**66,684**	**5.4%**	**26,905**	**2.2%**	**32,358**	**2.6%**
Argentina	2,932	4.3%	1,696	2.5%	1,695	2.5%
Bolivia	606	6.1%	164	1.7%	232	2.3%
Brazil	49,194	6.2%	11,620	1.5%	19,812	2.5%
Chile	7,362	3.8%	11,005	5.7%	8,883	4.6%
Colombia	2,799	10.2%	1,206	4.4%	882	3.2%
Ecuador	-1,480	-6.7%	485	2.2%	146	0.7%
French Guiana	12	2.1%	18	3.1%	15	2.5%
Guyana	428	11.8%	197	5.4%	155	4.3%
Paraguay	2,098	7.6%	546	2.0%	566	2.1%
Peru	1,928	7.0%	652	2.4%	541	2.0%
Suriname	-964	-24.6%	-179	-4.6%	-615	-15.7%
Uruguay	204	0.5%	-1,187	-2.9%	-484	-1.2%
Venezuela, Boliv Rep of	1,564	8.3%	683	3.6%	532	2.8%
**ASIA**	**348**	**0.0%**	**-37,784**	**-0.8%**	**-104,009**	**-2.1%**
Afghanistan	-7,769	-69.6%	-3,168	-28.4%	-5,037	-45.1%
Bahrain	3	11.4%	1	3.9%	1	3.1%
Bangladesh	5,329	9.7%	1,449	2.6%	1,258	2.3%
Bhutan	1,071	9.6%	323	2.9%	277	2.5%

Brunei Darussalam	-9	-3.1%	10	3.4%	8	2.5%
Cambodia	1,317	6.3%	508	2.4%	480	2.3%
China	103,502	4.6%	-17,115	-0.8%	-43,616	-1.9%
Cyprus	0	-0.6%	3	6.4%	2	5.0%
Maldives	4	10.5%	1	2.7%	0	1.4%
India	-24,009	-2.2%	3,528	0.3%	8,928	0.8%
Indonesia	-22,491	-5.1%	12,703	2.9%	-7,588	-1.7%
Iran, Islamic Rep of	-712	-5.4%	704	5.4%	374	2.9%
Iraq	-7	-0.4%	-77	-4.9%	-118	-7.4%
Israel	-168	-41.5%	27	6.7%	19	4.8%
Japan	-5,586	-4.0%	6,772	4.9%	5,100	3.7%
Jordan	77	10.4%	12	1.7%	9	1.3%
Korea, Dem People's Rep	2,086	10.1%	-1,699	-8.3%	673	3.3%
Korea, Republic of	-1,951	-7.7%	1,812	7.1%	1,335	5.2%
Kuwait	4	10.3%	1	2.7%	1	1.5%
Laos	2,880	11.4%	1,255	5.0%	993	3.9%
Lebanon	-196	-49.9%	-38	-9.6%	-45	-11.4%
Timor-Leste	19	10.4%	5	2.8%	4	2.3%
Malaysia	-26,149	-35.9%	2,781	3.8%	-279	-0.4%
Mongolia	206	10.6%	71	3.6%	64	3.3%
Myanmar	16,930	17.3%	2,364	2.4%	-180	-0.2%
Nepal	2,486	7.5%	821	2.5%	680	2.1%
Oman	5	4.7%	0	0.2%	-1	-1.1%
Pakistan	-3,306	-3.4%	-1,002	-1.0%	-2,872	-2.9%
Philippines	4,118	8.9%	1,874	4.1%	1,402	3.0%
Qatar	3	14.0%	-10	-51.9%	0	0.0%
Saudi Arabia	59	7.9%	13	1.8%	13	1.8%
Singapore	3	10.2%	1	2.7%	1	2.4%
Sri Lanka	312	2.5%	444	3.6%	364	3.0%
Syrian Arab Republic	-94	-24.8%	-21	-5.5%	-102	-26.8%
Thailand	-15,273	-11.1%	-14,800	-10.7%	-16,180	-11.7%
Turkey	-16,553	-11.7%	-44,567	-31.6%	-54,830	-38.9%
United Arab Emirates	5	8.7%	-21	-33.8%	-21	-34.6%
Viet Nam	-17,008	-12.3%	7,246	5.2%	4,556	3.3%
Yemen	101	8.9%	9	0.8%	26	2.3%
Armenia	239	7.7%	82	2.6%	71	2.3%
Azerbaijan, Republic of	59	6.8%	20	2.4%	17	2.0%
Georgia	-389	-19.7%	75	3.8%	53	2.7%
Kazakhstan	364	12.9%	-235	-8.3%	124	4.4%
Kyrgyzstan	94	8.7%	-85	-7.8%	-85	-7.9%
Tajikistan	732	9.7%	136	1.8%	109	1.4%
Turkmenistan	0	-3.3%	0	-18.0%	-1	-19.8%
Uzbekistan	10	11.1%	4	4.2%	3	3.4%
**OCEANIA**	**-17,468**	**-8.3%**	**16,592**	**7.9%**	**12,289**	**5.8%**
Australia	-21,707	-19.7%	9,932	9.0%	7,429	6.7%
Cook Islands	0	9.9%	0	8.0%	0	1.3%
Fiji Islands	-319	-13.3%	171	7.1%	117	4.9%
French Polynesia	1	9.2%	1	5.4%	0	2.6%
New Caledonia	-3	-6.1%	3	6.3%	2	4.9%
New Zealand	1,001	1.6%	4,445	6.9%	3,334	5.2%
Papua New Guinea	3,875	17.3%	1,820	8.1%	1,398	6.2%

Samoa	12	13.3%	5	5.3%	4	4.4%
Solomon Islands	-368	-3.6%	194	1.9%	-11	-0.1%
Tonga	1	13.2%	0	5.4%	0	-1.4%
Vanuatu	38	11.7%	21	6.5%	16	5.0%
**EUROPE**	**250,827**	**12.1%**	**86,234**	**4.2%**	**99,597**	**4.8%**
Albania	158	11.6%	159	11.7%	142	10.4%
Austria	-3,418	-6.9%	1,489	3.0%	663	1.3%
Belgium	265	5.4%	131	2.7%	109	2.2%
Bosnia and Herzegovina	164	1.3%	-1,390	-11.2%	-2,113	-17.0%
Bulgaria	2,132	15.2%	1,471	10.5%	1,114	8.0%
Croatia	965	10.2%	914	9.7%	697	7.4%
Czechia	3,967	10.3%	2,228	5.8%	1,636	4.2%
Denmark	899	17.6%	406	8.0%	310	6.1%
Finland	32,386	13.4%	18,076	7.5%	13,710	5.7%
France	11,412	13.9%	5,892	7.2%	4,051	4.9%
Germany	24,455	15.5%	11,017	7.0%	8,394	5.3%
Greece	-264	-10.6%	213	8.5%	168	6.8%
Hungary	1,939	20.5%	967	10.2%	728	7.7%
Luxembourg	75	12.6%	43	7.2%	31	5.2%
Ireland	444	5.2%	58	0.7%	-76	-0.9%
Italy	-2,241	-8.9%	1,872	7.5%	1,485	5.9%
Montenegro	-432	-22.6%	168	8.8%	131	6.9%
Netherlands	154	7.8%	108	5.5%	92	4.7%
North Macedonia	-122	-10.6%	37	3.2%	25	2.2%
Norway	5,866	14.3%	1,623	4.0%	674	1.6%
Poland	5,156	6.6%	1,070	1.4%	-8	0.0%
Portugal	-8,372	-16.8%	1,531	3.1%	743	1.5%
Romania	7,054	15.5%	3,760	8.3%	2,776	6.1%
Slovakia	1,660	10.7%	896	5.8%	665	4.3%
Slovenia	-497	-6.4%	696	8.9%	527	6.8%
Spain	458	1.0%	4,470	9.5%	3,233	6.8%
Sweden	35,242	12.5%	18,975	6.7%	14,218	5.0%
Switzerland	321	2.5%	579	4.5%	336	2.6%
United Kingdom	3,450	10.9%	1,407	4.4%	805	2.5%
Serbia	1,444	13.9%	733	7.1%	554	5.3%
Belarus	11,605	19.9%	5,618	9.7%	4,216	7.2%
Estonia	4,670	19.2%	2,366	9.7%	1,796	7.4%
Latvia	5,510	17.3%	3,056	9.6%	2,289	7.2%
Lithuania	2,915	19.9%	1,452	9.9%	1,102	7.5%
Moldova, Republic of	292	30.9%	76	8.0%	67	7.1%
Russian Federation	91,548	15.1%	-9,933	-1.6%	30,969	5.1%
Ukraine	9,567	24.7%	4,002	10.3%	3,337	8.6%
**WORLD**	**670,916**	**5.4%**	**249,829**	**2.0%**	**192,435**	**1.5%**

[1] BF = Biological-focus scenario; [2] AF = Agroeconomic-focus scenario; [3] BAC = Biological-agroeconomic compromise scenario.

Supplementary Table 2. Projected changes in world prices (%) of roundwood and manufactured wood products in 30x30 scenarios in 2030, relative to projected 2030 reference levels.

	**BF[1]**	**AF[2]**	**BAC[3]**
**Country/Region**	**Change in 2030 from REF (%)**		
Industrial roundwood	7.2	3.3	2.6
Fuelwood	12.2	3.4	3.2
Other Industrial roundwood	10.7	6.5	5.0
**Average all roundwood**	**9.6**	**4.5**	**3.7**
**Sawnwood**	**3.0**	**2.4**	**1.6**
Plywood/Veneer	2.4	1.3	0.8
Particleboard	1.8	1.0	0.8
Fiberboard	1.8	1.0	0.8
**Average wood-based panels**	**2.1**	**1.1**	**0.8**
Newsprint	4.3	0.5	0.3
Printing and writing paper	0.7	0.4	0.3
Other paper and paperboard	0.5	0.3	0.2
**Average all paper and paperboard**	**1.4**	**0.4**	**0.3**

[1] BF = Biological-focus scenario; [2] AF = Agroeconomic-focus scenario; [3] BAC = Biological-agroeconomic compromise scenario.

Supplementary Table 3. Percent changes in cumulative consumption and production of roundwood, finished solidwood products, and paper products in 30x30 scenarios relative to reference, 2025-2060.

**Region**	**Roundwood**		**Finished solidwood**		**Finished paper**	
	**Production**	**Consumption**	**Production**	**Consumption**	**Production**	**Consumption**
**AFRICA**						
BF[1]	-2.93%	-2.43%	-9.49%	-3.30%	-0.84%	-0.94%
AF[2]	-0.50%	-0.43%	-1.58%	-1.06%	-0.71%	-0.37%
BAC[3]	-0.49%	-0.33%	-1.94%	-0.91%	-0.72%	-0.26%
**N/C AMERICA**						
BF	2.93%	2.98%	6.03%	-1.26%	2.80%	-0.65%
AF	1.01%	0.96%	2.28%	-0.75%	0.96%	-0.32%
BAC	2.93%	2.98%	6.03%	-1.26%	2.80%	-0.65%
**S AMERICA**						
BF	-1.31%	-1.35%	-4.14%	-1.94%	-0.18%	-0.61%
AF	-0.80%	-1.50%	-4.19%	-0.90%	-1.17%	-0.31%
BAC	0.08%	-0.42%	-0.98%	-0.64%	-0.67%	-0.23%
**ASIA**						
BF	-5.48%	-3.00%	-1.79%	-1.48%	-2.40%	-0.61%
AF	-2.76%	-0.60%	0.12%	-0.89%	-0.58%	-0.25%
BAC	-3.29%	-1.12%	-1.07%	-0.80%	-0.46%	-0.18%
**OCEANIA**						
BF	-13.38%	-4.31%	-3.58%	-1.55%	-7.45%	-0.96%
AF	3.10%	-0.74%	0.47%	-0.72%	-6.10%	-0.39%
BAC	2.26%	-0.64%	0.18%	-0.53%	-4.77%	-0.27%
**EUROPE**						
BF	2.02%	-3.14%	-3.90%	-1.40%	1.00%	-0.76%
AF	-0.83%	-3.79%	-3.51%	-0.78%	-0.12%	-0.33%
BAC	0.88%	-2.18%	-2.24%	-0.56%	-0.12%	-0.23%
**WORLD**						
BF	-1.84%	-1.84%	-1.48%	-1.48%	-0.66%	-0.66%
AF	-1.04%	-1.04%	-0.84%	-0.84%	-0.28%	-0.28%
BAC	-0.69%	-0.69%	-0.70%	-0.70%	-0.20%	-0.20%

[1] BF = Biological-focus scenario; [2] AF = Agroeconomic-focus scenario; [3] BAC = Biological-agroeconomic compromise scenario.

Supplementary Table 4. Number and proportion of countries with positive changes in cumulative Net Output Values in 30x30 scenarios, 2025-2060.

**Regions**	**Total countries**	**No. of countries with positive NOVs**			**% of countries with positive NOVs**		
		**BF[1]**	**AF[2]**	**BAC[3]**	**BF**	**AF**	**BAC**
AFRICA	50	38	46	44	76%	92%	88%
N/C AMERICA	22	16	21	20	73%	95%	91%
S AMERICA	13	11	11	11	85%	85%	85%
ASIA	47	28	33	32	60%	70%	68%
OCEANIA	11	7	11	9	64%	100%	82%
EUROPE	37	30	35	34	81%	95%	92%
WORLD	180	130	157	150	72%	87%	83%

[1] BF = Biological-focus scenario; [2] AF = Agroeconomic-focus scenario; [3] BAC = Biological-agroeconomic compromise scenario.

Supplementary Table 5. Estimated mean harvest costs and assumed increases in those costs under two sensitivity tests.

Region	Harvest cost † ($/m³)	Transport cost ($/m³)	Total harvest cost ($/m³)	Assumed increases in transport costs for sensitivity test			
				25%		12.5%	
				Total harvest cost ($/m³)	Implied variation (%)	Total harvest cost ($/m³)	Implied variation (%)
Latin America and Caribbean ‡	6.98	3.90	10.88	11.85	9.0	11.36	4.5
Uruguay	12.90	7.20	20.10	21.90	9.0	21.00	4.5
Asia §	9.19	5.13	14.32	15.60	9.0	14.96	4.5
North America	15.17	8.47	23.63	25.75	9.0	24.69	4.5
Australia	21.45	11.97	33.42	36.41	9.0	34.91	4.5
Western Europe	30.14	16.82	46.96	51.16	9.0	49.06	4.5
Eastern Europe	30.54	17.04	47.58	51.84	9.0	49.71	4.5
Europe Average	30.34	16.93	47.27	51.50	9.0	49.39	4.5
Africa	11.43	6.38	17.80	19.40	9.0	18.60	4.5

Note: All costs are adjusted to 2018-constant-dollar values.

† Includes pre-harvesting and harvesting costs.

‡ Excludes Uruguay.

§ Especially SE Asia; excludes outlier countries with very high costs.

Supplementary Table 6. Effects of a higher price elasticity on Net Output Values (billion 2018 US $, cumulative, 2025-2060) in world regions in 30x30 scenarios relative to reference scenario. Top rows: NOVs with mean cost; bottom rows: NOVs with 4.5% and 9% higher and lower costs than mean harvest costs.

	**Change relative to REF (Cumulative billion 2018 US $)**			**% Change relative to REF**				**Change relative to REF (Cumulative billion 2018 US $)**			**% Change relative to REF**		
**Base cost**	**BF[1]**	**AF[2]**	**BAC[3]**	**BF**	**AF**	**BAC**		**BF**	**AF**	**BAC**	**BF**	**AF**	**BAC**
AFRICA	70.54	24.88	16.83	4.5%	1.6%	1.1%							
N/C AMERICA	231.91	90.45	100.14	9.3%	3.6%	4.0%							
S AMERICA	55.65	20.90	26.49	4.5%	1.7%	2.1%							
ASIA	-52.23	-81.71	-137.44	-1.1%	-1.7%	-2.8%							
OCEANIA	-20.93	13.66	10.08	-10.0%	6.5%	4.8%							
EUROPE	217.62	65.50	85.81	10.5%	3.2%	4.2%							
WORLD	502.56	133.67	101.92	4.0%	1.1%	0.8%							
	**Effects of higher harvest cost**							**Effects of lower harvest cost**					
**4.5% higher cost**	**BF**	**AF**	**BAC**	**BF**	**AF**	**BAC**	**4.5% lower cost**	**BF**	**AF**	**BAC**	**BF**	**AF**	**BAC**
AFRICA	45.53	-0.81	-8.85	2.9%	-0.1%	-0.6%	AFRICA	95.55	50.56	42.52	6.2%	3.3%	2.7%
N/C AMERICA	197.66	56.81	66.25	7.9%	2.3%	2.6%	N/C AMERICA	266.17	124.10	134.03	10.6%	5.0%	5.4%
S AMERICA	47.64	12.85	18.38	3.9%	1.0%	1.5%	S AMERICA	63.66	28.95	34.61	5.2%	2.3%	2.8%
ASIA	-89.48	-119.97	-175.51	-1.8%	-2.4%	-3.6%	ASIA	-14.98	-43.44	-99.37	-0.3%	-0.9%	-2.0%
OCEANIA	-24.87	8.96	5.42	-11.8%	4.3%	2.6%	OCEANIA	-16.99	18.35	14.75	-8.1%	8.7%	7.0%
EUROPE	141.12	-8.95	10.03	6.8%	-0.4%	0.5%	EUROPE	294.11	139.95	161.59	14.2%	6.8%	7.8%
WORLD	317.60	-51.12	-84.29	2.5%	-0.4%	-0.7%	WORLD	687.52	318.47	288.13	5.5%	2.6%	2.3%
**9% higher cost**	**BF**	**AF**	**BAC**	**BF**	**AF**	**BAC**	**9% lower cost**	**BF**	**AF**	**BAC**	**BF**	**AF**	**BAC**
AFRICA	20.52	-26.50	-34.54	1.3%	-1.7%	-2.2%	AFRICA	120.56	76.25	68.21	7.8%	4.9%	4.4%
N/C AMERICA	163.41	23.17	32.36	6.5%	0.9%	1.3%	N/C AMERICA	300.42	157.74	167.92	12.0%	6.3%	6.7%
S AMERICA	39.64	4.80	10.27	3.2%	0.4%	0.8%	S AMERICA	71.66	37.00	42.72	5.8%	3.0%	3.5%
ASIA	-126.74	-158.24	-213.58	-2.6%	-3.2%	-4.4%	ASIA	22.28	-5.17	-61.30	0.5%	-0.1%	-1.2%
OCEANIA	-28.82	4.26	0.75	-13.7%	2.0%	0.4%	OCEANIA	-13.05	23.05	19.41	-6.2%	11.0%	9.2%
EUROPE	64.63	-83.41	-65.75	3.1%	-4.0%	-3.2%	EUROPE	370.61	214.40	237.37	17.9%	10.4%	11.5%
WORLD	132.64	-235.92	-270.50	1.1%	-1.9%	-2.2%	WORLD	872.48	503.27	474.34	7.0%	4.0%	3.8%

[1] BF = Biological-focus scenario; [2] AF = Agroeconomic-focus scenario; [3] BAC =Biological-agroeconomic compromise scenario.

Supplementary Table 7. Estimated changes in Net Output Values (billion 2018 US $, cumulative, 2025-2060) in world regions in 30x30 scenarios relative to reference scenario, using base price elasticity. Top rows: NOVs with mean cost; bottom rows: NOVs with 4.5% and 9.5% higher and lower costs than mean harvest costs.

	Change relative to REF (Cumulative billion 2018 US $)			% Change relative to REF				Change relative to REF (Cumulative billion 2018 US $)			% Change relative to REF		
**Base cost**	**BF[1]**	**AF[2]**	**BAC[3]**	**BF**	**AF**	**BAC**		**BF**	**AF**	**BAC**	**BF**	**AF**	**BAC**
AFRICA	98.55	41.23	31.37	6.4%	2.7%	2.0%							
N/C AMERICA	271.97	116.65	120.83	10.9%	4.7%	4.8%							
S AMERICA	66.68	26.91	32.36	5.4%	2.2%	2.6%							
ASIA	0.35	-37.78	-104.01	0.0%	-0.8%	-2.1%							
OCEANIA	-17.47	16.59	12.29	-8.3%	7.9%	5.8%							
EUROPE	250.83	86.23	99.60	12.1%	4.2%	4.8%							
WORLD	670.92	249.83	192.44	5.4%	2.0%	1.5%							
				**Effects of higher harvest cost**							**Effects of lower harvest cost**		
**4.5% higher cost**	**BF**	**AF**	**BAC**	**BF**	**AF**	**BAC**	**4.5% lower cost**	**BF**	**AF**	**BAC**	**BF**	**AF**	**BAC**
AFRICA	73.55	15.60	5.74	4.7%	1.0%	0.4%	AFRICA	123.56	66.86	57.00	8.0%	4.3%	3.7%
N/C AMERICA	237.86	83.17	87.11	9.5%	3.3%	3.5%	N/C AMERICA	306.09	150.13	154.55	12.2%	6.0%	6.2%
S AMERICA	58.73	18.91	24.29	4.8%	1.5%	2.0%	S AMERICA	74.64	34.90	40.43	6.0%	2.8%	3.3%
ASIA	-36.77	-75.97	-141.98	-0.7%	-1.5%	-2.9%	ASIA	37.46	0.40	-66.04	0.8%	0.0%	-1.3%
OCEANIA	-21.40	11.92	7.65	-10.2%	5.7%	3.6%	OCEANIA	-13.54	21.27	16.93	-6.4%	10.1%	8.0%
EUROPE	174.93	12.46	24.55	8.5%	0.6%	1.2%	EUROPE	326.72	160.00	174.64	15.8%	7.7%	8.5%
WORLD	486.90	66.10	7.36	3.9%	0.5%	0.1%	WORLD	854.93	433.56	377.51	6.9%	3.5%	3.0%
**9% higher cost**	**BF**	**AF**	**BAC**	**BF**	**AF**	**BAC**	**9% lower cost**	**BF**	**AF**	**BAC**	**BF**	**AF**	**BAC**
AFRICA	48.54	-10.03	-19.89	3.1%	-0.6%	-1.3%	AFRICA	148.56	92.49	82.63	9.6%	6.0%	5.3%
N/C AMERICA	203.74	49.69	53.40	8.1%	2.0%	2.1%	N/C AMERICA	340.20	183.61	188.26	13.6%	7.3%	7.5%
S AMERICA	50.78	10.92	16.22	4.1%	0.9%	1.3%	S AMERICA	82.59	42.89	48.49	6.7%	3.5%	3.9%
ASIA	-73.88	-114.15	-179.95	-1.5%	-2.3%	-3.7%	ASIA	74.58	38.58	-28.07	1.5%	0.8%	-0.6%
OCEANIA	-25.33	7.24	3.01	-12.0%	3.4%	1.4%	OCEANIA	-9.61	25.94	21.57	-4.6%	12.3%	10.3%
EUROPE	99.04	-61.31	-50.49	4.8%	-3.0%	-2.4%	EUROPE	402.61	233.78	249.69	19.5%	11.3%	12.1%
WORLD	302.89	-117.63	-177.71	2.4%	-0.9%	-1.4%	WORLD	1,038.94	617.29	562.58	8.3%	5.0%	4.5%

[1] BF = Biological-focus scenario; [2] AF = Agroeconomic-focus scenario; [3] BAC =Biological-agroeconomic compromise scenario.

Supplementary Table 8. Effects of a lower price elasticity of supply and higher and lower harvest costs on estimated Net Output Values (NOV) in major world regions in 30x30 scenarios relative to reference scenario. Top rows: NOVs with mean cost; bottom rows: NOVs with 4.5% and 9% higher and lower costs.

	**Change relative to REF (Cumulative billion 2018 US $)**			**% Change relative to REF**				**Change relative to REF (Cumulative billion 2018 US $)**			**% Change relative to REF**		
**Base cost**	**BF[1]**	**AF[2]**	**BAC[3]**	**BF**	**AF**	**BAC**		**BF**	**AF**	**BAC**	**BF**	**AF**	**BAC**
AFRICA	135.51	61.83	51.58	8.7%	4.0%	3.3%							
N/C AMERICA	323.97	153.82	155.50	13.0%	6.2%	6.2%							
S AMERICA	83.31	37.21	41.19	6.7%	3.0%	3.3%							
ASIA	73.88	17.86	-53.77	1.5%	0.4%	-1.1%							
OCEANIA	-13.39	20.57	15.91	-6.4%	9.8%	7.6%							
EUROPE	298.52	118.80	128.05	14.5%	5.8%	6.2%							
WORLD	901.80	410.09	338.46	7.2%	3.3%	2.7%							
	**Effects of higher harvest cost**							**Effects of lower harvest cost**					
**4.5% higher cost**	**BF**	**AF**	**BAC**	**BF**	**AF**	**BAC**	**4.5% higher cost**	**BF**	**AF**	**BAC**	**BF**	**AF**	**BAC**
AFRICA	110.58	36.28	26.02	7.1%	2.3%	1.7%	AFRICA	160.44	87.39	77.14	10.3%	5.6%	5.0%
N/C AMERICA	290.03	120.50	121.94	11.6%	4.8%	4.9%	N/C AMERICA	357.92	187.13	189.05	14.3%	7.5%	7.6%
S AMERICA	75.41	29.27	33.18	6.1%	2.4%	2.7%	S AMERICA	91.21	45.15	49.20	7.4%	3.7%	4.0%
ASIA	36.93	-20.21	-91.63	0.8%	-0.4%	-1.9%	ASIA	110.84	55.94	-15.91	2.3%	1.1%	-0.3%
OCEANIA	-17.30	15.92	11.30	-8.2%	7.6%	5.4%	OCEANIA	-9.48	25.22	20.53	-4.5%	12.0%	9.8%
EUROPE	223.32	45.69	53.73	10.8%	2.2%	2.6%	EUROPE	373.71	191.92	202.38	18.1%	9.3%	9.8%
WORLD	718.97	227.44	154.54	5.8%	1.8%	1.2%	WORLD	1,084.63	592.75	522.38	8.7%	4.8%	4.2%
**9% higher cost**	**BF**	**AF**	**BAC**	**BF**	**AF**	**BAC**	**9% higher cost**	**BF**	**AF**	**BAC**	**BF**	**AF**	**BAC**
AFRICA	85.65	10.73	0.46	5.5%	0.7%	0.0%	AFRICA	185.37	112.94	102.71	11.9%	7.3%	6.6%
N/C AMERICA	256.08	87.19	88.39	10.2%	3.5%	3.5%	N/C AMERICA	391.87	220.45	222.60	15.7%	8.8%	8.9%
S AMERICA	67.52	21.33	25.17	5.5%	1.7%	2.0%	S AMERICA	99.10	53.09	57.21	8.0%	4.3%	4.6%
ASIA	-0.03	-58.29	-129.49	0.0%	-1.2%	-2.6%	ASIA	147.79	94.02	21.95	3.0%	1.9%	0.4%
OCEANIA	-21.21	11.26	6.68	-10.1%	5.4%	3.2%	OCEANIA	-5.58	29.87	25.14	-2.7%	14.2%	12.0%
EUROPE	148.13	-27.43	-20.60	7.2%	-1.3%	-1.0%	EUROPE	448.91	265.04	276.70	21.7%	12.8%	13.4%
WORLD	536.14	44.79	-29.39	4.3%	0.4%	-0.2%	WORLD	1,267.46	775.40	706.30	10.2%	6.2%	5.7%

[1] BF = Biological-focus scenario; [2] AF = Agroeconomic-focus scenario; [3] BAC =Biological-agroeconomic compromise scenario.

Supplementary References

1. Waldron, A. *et al.* Protecting 30% of the planet for nature: costs, benefits and economic implications. 58 https://www.conservation.cam.ac.uk/files/waldron_report_30_by_30_publish.pdf (2020).
2. Jung, M. *et al.* Areas of global importance for conserving terrestrial biodiversity, carbon and water. *Nature Ecology & Evolution* **5**, 1499–1509 (2021).
3. Leclère, D. *et al.* Supporting material for the article entitled "Bending the curve of terrestrial biodiversity needs an integrated strategy. International Institute for Applied Systems Analysis. https://pure.iiasa.ac.at/id/eprint/17565/. (2019).
4. Leclère, D. *et al.* Bending the curve of terrestrial biodiversity needs an integrated strategy. *Nature* **585**, 551–556 (2020).
5. Nepal, P., Buongiorno, J., Johnston, C. M. T., Prestemon, J. P. & Guo, J. Global forest products trade model. in *International trade in forest products: Lumber trade disputes, models and examples* (eds van Kooten, G. C. & Voss, L.) 110–141 (CABI, 2021).
6. Buongiorno, J. Global modelling to predict timber production and prices: The GFPM approach. *Forestry: An International Journal of Forest Research* **88**, 291–303 (2015).
7. Buongiorno, J. & Johnston, C. Potential effects of US protectionism and trade wars on the global forest sector. *Forest Science* **64**, 121–128 (2018).
8. Johnston, C. M. T. & Radeloff, V. C. Global mitigation potential of carbon stored in harvested wood products. *Proceedings of the National Academy of Sciences of the United States of America* **116**, 4526–14531 (2019).

9. Johnston, C. M. T. & Buongiorno, J. Impact of Brexit on the forest products industry of the United Kingdom and the rest of the world. *Forestry: An International Journal of Forest Research* **90**, 47–57 (2017).
10. Buongiorno, J., Zhu, S., Zhang, D., Turner, J. & Tomberlin, D. *The Global Forest Products Model: Structure, Estimation, and Applications*. (Elsevier, 2003).
11. Turner, J. A., Buongiorno, J. & Zhu, S. An economic model of international wood supply, forest stock and forest area change. *Scandinavian Journal of Forest Research* **21**, 73–86 (2006).
12. Samuelson, P. A. Spatial price equilibrium and linear programming. *American Economic Review* **42**, 283-301. (1952).
13. Takayama, T., Hashimoto, H. & Uri, N. D. Spatial and temporal price and allocation modeling: Some extensions. *Socio-Economic Planning Sciences* **18**, 227–234 (1984).
14. Li, B. *et al.* An improved global land cover mapping in 2015 with 30 m resolution (GLC-2015) based on a multisource product-fusion approach. *Earth System Science Data* **15**, 2347–2373 (2023).
15. Jung, M. *et al.* The global exposure of species ranges and protected areas to forest management. *Diversity and Distributions* **28**, 1487–1496 (2022).
16. FAO. *Global Forest Resources Assessment 2020. Main Report.* (FAO, Rome, 2020).
17. Koop, G. & Tole, L. Is there an environmental Kuznets curve for deforestation? *Journal of Development Economics* **58**, 231–244 (1999).
18. Just, R. E. & Hueth, D. L. Welfare measures in a multimarket framework. *American Economic Review* **69**, 947–954 (1979).

19. Thurman, W. N. & Easley, J. E. Valuing changes in commercial fishery harvests: A general equilibrium derived demand analysis. *Journal of Environmental Economics and Management* **22**, 226–240 (1992).
