# Supplementary material for: Economic costs of global forest protection may be overstated
Source: Nat Commun. 2026 May 20;17:6649. doi: 10.1038/s41467-026-73569-0 (PMC13381589; doi:10.1038/s41467-026-73569-0)
Supplement: Supplementary file 2 — Description of Additional Supplementary Files [file 41467_2026_73569_MOESM2_ESM.pdf]

Description of Additional Supplementary Files:

Supplementary Data 1: Forest area changes by 2030 in reference and alternate protected forest area scenarios

Supplementary Data 2: Projected main results for reference and alternate protected forest area scenarios

Supplementary Data 3: Results of the sensitivity analysis-high supply price elasticities

Supplementary Data 4: Results of the sensitivity analysis-high and low production costs

Supplementary Data 5: Results of the sensitivity analysis-low supply price elasticities
