# Supplementary material for: Economic costs of global forest protection may be overstated
Source: Nat Commun. 2026 May 20;17:6649. doi: 10.1038/s41467-026-73569-0 (PMC13381589; doi:10.1038/s41467-026-73569-0)
Supplement: Supplementary file 9 — Transparent Peer Review file [file 41467_2026_73569_MOESM9_ESM.pdf]

nature portfolio

Peer Review File

Economic costs of global forest protection may be overstated

Corresponding Author: Dr Prakash Nepal

This file contains all reviewer reports in order by version, followed by all author rebuttals in order by version.

Version 0:

Reviewer comments:

Reviewer #1

(Remarks to the Author)
I reviewed the manuscript "Reports of the economic cost of global forest protection have been greatly exaggerated" submitted to Nature Communications for publication.
It present exciting and highly relevant work on the global and regional costs of forest protection. It finds that costs of conservation derived from the estimation of opportunity costs depend very much on the method chosen. The authors use a partial equilibrium model that allows for accounting of economic effects when market react to a reduction of forest area available for wood supply. This method results in much lower estimated costs compared to methods that are summing up opportunity costs that have been estimated at regional or local level.
The manuscript builds on grey literature published and makes reference to other modelling work. My observation is that this list could be expanded more, especially when it comes to a comparison to regional studies (see example provided below).
The manuscript is very well written. The authors explain the applied methodology and their findings in a clear and comprehensive manner that makes it easy for non-experts of the method to follow their approach. A few smaller clarifications needed are listed below.
The scenario names are not very intuitive. The authors picked names that aim to provide a generic character of the scenarios (e.g. "Crown Jewels"). However, when readers study figures and tables these names do not help to understand what the scenario is actually about. Names that are more clearly linked to the changes in assumptions in the scenarios would also help following the discussion.
There are two medium flaws that I identified:
1) The results clearly show that there are large differences between global results and regional level outcomes. The main conclusion of the authors is that global modeling approaches that integrate market effects are more appropriate for estimating opportunity costs. This implies that regional studies are of less value for policy making and bear the risk of exaggerating opportunity costs. However, there is little discussion provided by the authors with concrete comparisons of regional versus global studies. Here, it would be useful if the literature referred to could be expanded. For example, a critical discussion with findings of higher opportunity costs like presented by Regelmann et al. or similar studies would increase the value of the study for policy makers. The authors could get more explicit on how global models that lack the specificity of national or regional forest situations are giving better advice to policy makers regarding opportunity costs of conservation. The manuscript is not yet very convincing here.
Regelmann C, Rosenkranz L, Seintsch B, Dieter M (2023) Economic evaluation of different implementation variants and categories of the EU Biodiversity Strategy 2030 using forestry in Germany as a case study. Forests 14(6):1173, DOI:10.3390/f14061173
2) An important aspect of the costs of conservation are avoided costs of damage to ecosystems. Avoided costs or the value of the ecosystem services that would have been lost if an area was not protected are as relevant as opportunity costs of the protection. The manuscript is not making reference to avoided costs. I think it is necessary to put both concepts into relation. I assume that avoided costs would be an additional item in the cost-benefit analysis. How would this change the results? I understand that this requires additional data different from what the authors have collected. However, there should be at least some brief discussion on the approach and how it contributes to more appropriate estimates of cost-benefit analyses of forest conservation.
The authors mention that it is likely that there will be substitution of wood products by other material (line 129). However, they do not state at all whether they consider such effects, what difference it would make and how such effects could be included.
Some minor errors or lack of clarity was noticed at the following points of the manuscript:

- Line 7: term “30x30” should be explained in the abstract
- Line 59: Seems there is a second verb missing in the sentence starting “We explain…”.
- Line 72: GFPM is mentioned here for the first time and needs to be introduced
- Lines 124 and 136: I find the use of the unit “per country” not very useful. What is the reason?
- Lines 257-259: Repetition of first lines of paragraph above “The GFPM generates…”
- Fig1-3: Provide an explanation of the scenario abbreviation in figure captions that describe the character of the scenario
- Tab S5-S7: Please revise table captions. It is not clear to the reader what Tab S6 presents in difference to Tab S5. Make the table captions fully consistent that from one glance the differences between the tables are clear.

Hannes Böttcher

Reviewer #2

(Remarks to the Author)
The angle of the manuscript is refreshing. It is intuitive but has been overlooked in previous studies. I find the methodology to be solid and the analysis, in general, rigorous. However, I think the framing of the results is problematic and needs further work.

My biggest concern is how “economic cost” is defined in the manuscript. The analysis focuses on net output value (NOV) for producers rather than on economic welfare more broadly. While NOV is a valid metric, interpreting increases in NOV as evidence of low economic costs is potentially misleading. To assess the big picture, impacts on the consumer side (e.g., welfare losses due to price increases and downstream effects) needs to be addressed more explicitly.

A related concern is that substitution effects are treated rather lightly. While substitution away from wood products is mentioned in the discussion, it is not explored to the extent warranted, given the long time horizon and the important role of price effects in driving the changes.

The results show substantial distributional heterogeneity across countries, but this is largely glossed over in the discussion. Given the policy relevance of 30x30, these distributional effects deserve more attention, particularly if the authors are aiming for global policy-relevance.

To summarize, I think the results are noteworthy, and the analysis fills an important gap in the literature. The methodology itself is standard for the forest economics field and sound (no original contribution, though), but the interpretation and conclusions would benefit from more careful framing and stronger alignment with what the analysis actually demonstrates. The work appears reproducible. Once again, the claims would be more convincing with a clearer distinction between producer outcomes and broader economic impacts/welfare.

Reviewer #3

(Remarks to the Author)
The manuscript "Reports of the economic cost of global forest protection have been greatly exaggerated" is an important addition to the literature examining the economic costs of global protection of terrestrial ecosystems, particularly in the context of the "30 by 30" conservation target agreed to in the Kunming-Montreal Global Biodiversity Framework (KMGBF). There are few studies of this type in the literature even though the question of the costs of implementing the 30 x 30 target of the KMGBF are of utmost importance.

Overall, the analysis based on the state-of-the-art tools and does a commendable job in terms of the scenarios explored. As such, this manuscript provides strong arguments that some analyses of opportunity costs for large scale conservation of forests may have been greatly overestimated.

However, I have several misgivings about the paper, most of which would seem relatively simple to correct:

1) It is true that some studies have indicated that there could be high opportunity costs associated with conserving forest ecosystems at very large scales (primarily in the context of conserving them as carbon sinks). However, the authors overstate the case that analyses with models that account for economic feedbacks have not been undertaken, and that (without any nuance in the abstract) "protecting earth’s remaining forests...is thought to represent trillions of dollars of lost income". Several recent studies (albeit some are "grey literature") have concluded that the opportunity costs of implementing the 30 x 30 are quite low, or could even provide net benefits (see the very nice synthesis in Kedward & Poupard 2024). This includes the study (Waldron et al., citation 16) on which the forestry scenarios in this manuscript are based. It is only in the discussion that this and other work reaching similar conclusions is provided for context. The work in this manuscript is very important, but it isn't the first to come to similar conclusions and this should be made clear in the title, abstract and introduction.

2) Opportunity costs are not the only costs associated with massively increasing global protected areas. Other costs include, but are not limited to administration, enforcement, protected-area infrastructure, monitoring, etc. This analysis was not designed to account for these costs, but these costs could be very high and should be acknowledged. (Note that the title is

currently "Reports of the economic cost of global forest protection have been greatly exaggerated". This manuscript does not account for total economic cost).

3) The authors need to justify the sensitivity analysis for price elasticity. They state that "we adjusted the price elasticity parameter in either direction by +/- 15%". Why this range? This seems to be a relatively narrow range for a sensitivity analysis (see comments below on product substitution). Where are the references/justification for this? Increasing price elasiticity by much more that 15% could well result in a scenario where opportunity costs are high. Why wasn't this possibility explored? Without a strong justification for the range of elasticity explored in the manuscript, I would think it essential to extend the analysis to a much wider range of elasticities.

4) The authors should do a better job of highlighting the limitations of an analysis that uses a partial-equilibrium model that focuses exclusively on the forestry sector. For example, the analysis does not account for spillover effects on other sectors and markets such as agriculture and energy. As another example, this analysis doesn't account for material substitution. For example, if prices for forestry products rise substantially (which is an outcome of this analysis), consumers may find substitutes thereby reducing demand. This has been taken into account to some extent by examining elasticity, but this does not explicitly account for product substitution which may or may not be easily reversible. There are several other major limitations of a partial-equilibrium model that focuses exclusively on the forestry sector that mean that much greater caution is necessary when interpreting the results of this analysis. Overall, the discussion should be much more open about the limitations associated with the methods and assumptions.

Kedward, K., Poupard, A. (2024). The economic and financial risks of implementing the ‘30x30’ Global Biodiversity Framework targets. UCL Institute for Innovation and Public Purpose, Working Paper Series (IIPP WP 2024-13). https://www.ucl.ac.uk/bartlett/public-purpose/wp2024-13.

Version 1:

Reviewer comments:

Reviewer #1

(Remarks to the Author)
MY concerns as reviewer have been addressed by the authors.

Reviewer #2

(Remarks to the Author)
The concerns raised in the first round of review were primarily related to the framing and interpretation of results rather than the methodology and analysis. I believe that the authors have made substantive revisions in response to these concerns. In particular, they now clearly acknowledge that their measure of “economic cost” refers specifically to producer opportunity costs and does not capture broader welfare impacts, including consumer losses or avoided environmental damages. This clarification is important for improving transparency, although some risk of misinterpretation may still linger if the distinction is not consistently emphasized throughout the manuscript.

The discussion of substitution effects has also been improved. The authors now explicitly recognize that their modeling framework cannot capture cross-sectoral substitution (e.g., steel or concrete) and that this limitation may affect both economic and environmental outcomes. While this remains a limitation, the revised manuscript contextualizes the implications and appropriately frames results as conditional on limited substitution.

In addition, the authors have strengthened the discussion of distributional heterogeneity across countries. The revision highlights the policy relevance of uneven impacts and the need for differentiated policy responses.

Overall, the revisions address the main concerns raised in the first round of review.

Reviewer #3

(Remarks to the Author)
I'd like to thank the authors for thoroughly addressing the comments from the reviewers. Additions to the paper, particularly in the discussion, are very helpful in understanding the strengths and limitations of the analysis presented in the paper. The only issue where I find the response a bit weak is related to sensitivity of price elasticity. Perhaps a few additional words in the methods about the choice of +/- 15% would be helpful so at least readers can better understand the reasoning.

Open Access This Peer Review File is licensed under a Creative Commons Attribution 4.0 International License, which permits use, sharing, adaptation, distribution and reproduction in any medium or format, as long as you give appropriate credit to the original author(s) and the source, provide a link to the Creative Commons license, and indicate if changes were made.
In cases where reviewers are anonymous, credit should be given to 'Anonymous Referee' and the source.
The images or other third party material in this Peer Review File are included in the article's Creative Commons license, unless indicated otherwise in a credit line to the material. If material is not included in the article's Creative Commons license and your intended use is not permitted by statutory regulation or exceeds the permitted use, you will need to obtain permission directly from the copyright holder.
To view a copy of this license, visit https://creativecommons.org/licenses/by/4.0/

Response to the reviewers' comments

Manuscript # NCOMMS-25-84972

Response to Reviewer #1's comments

****

General comment 1: I reviewed the manuscript "Reports of the economic cost of global forest protection have been greatly exaggerated" submitted to Nature Communications for publication. It present exciting and highly relevant work on the global and regional costs of forest protection. It finds that costs of conservation derived from the estimation of opportunity costs depend very much on the method chosen. The authors use a partial equilibrium model that allows for accounting of economic effects when market react to a reduction of forest area available for wood supply. This method results in much lower estimated costs compared to methods that are summing up opportunity costs that have been estimated at regional or local level.

Response to general comment1: *Thank you for your thoughtful and detailed review. We appreciate your recognition of the relevance and novelty of our work.*

General comment 2: The manuscript builds on grey literature published and makes reference to other modelling work. My observation is that this list could be expanded more, especially when it comes to a comparison to regional studies (see example provided below).

Response to general comment 2: Thank you for this suggestion. In the revised version, we've incorporated additional relevant literature, including the example you provided. We added the following text in the introduction section*:*

"We note here that a few recent studies have found similar outcomes, i.e., these costs may be far lower than previously assumed, and in some cases even net beneficial once broader economic feedbacks, avoided damages, and ecosystem-service benefits are considered. For example, Kedward and Poupard[15] synthesize recent modelling exercises and report that the opportunity costs of implementing the 30×30 target are often modest, particularly when accounting for the value of ecosystem services, reduced climate related damages, and the stabilizing effects of conservation on long term economic systems. Similarly, Waldron et al.[16], whose scenarios underpin the conservation pathways examined here, report positive economic effects from expanding protected areas. However, their estimates reflect gross output changes in the forestry sector and do not deduct harvest and transport costs, making them unsuitable for assessing forestry sector net output values. Similarly, following Waldron et al.'s[16]

approach, Johnson et al.[17] estimated the cost of implementing 30x30 policies using an integrated ecosystem-economy model (GTAP-Invest) and found the total cost of such policies to be modest at $115 billion (0.1% of global GDP), and to be only $13 billion if the economic benefits of avoided CO_2 emissions were accounted for.

The present study builds on this literature by providing a net output perspective that explicitly incorporates production costs within a sectorally detailed modelling framework that captures projected shifts in prices, production, consumption and trade of roundwood because of increased forest protection. By focusing on net output value within the forestry sector, our analysis complements existing work and helps refine the understanding of how opportunity costs behave under large-scale forest protection scenarios."

General comment 3: The manuscript is very well written. The authors explain the applied methodology and their findings in a clear and comprehensive manner that makes it easy for non-experts of the method to follow their approach. A few smaller clarifications needed are listed below.

Response to general comment 3: Thank you for your positive feedback on the clarity and comprehensiveness of the manuscript. Below, we outline how each of the clarifications you suggested has been addressed in the revised version.

General comment 4: The scenario names are not very intuitive. The authors picked names that aim to provide a generic character of the scenarios (e.g. "Crown Jewels"). However, when readers study figures and tables these names do not help to understand what the scenario is actually about. Names that are more clearly linked to the changes in assumptions in the scenarios would also help following the discussion.

Response to general comment 4: Thank you for this feedback. In the revised manuscript, we have updated the scenario names and added table notes and figure captions to spell out the abbreviated scenario names as follows:

"AF = Agro-economic focus scenario.

BF = Biological-focus scenario.

BAC = Biological-agroeconomic compromise scenario."

General comment 5: There are two medium flaws that I identified:
1) The results clearly show that there are large differences between global results and regional level outcomes. The main conclusion of the authors is that global modeling

approaches that integrate market effects are more appropriate for estimating opportunity costs. This implies that regional studies are of less value for policy making and bear the risk of exaggerating opportunity costs. However, there is little discussion provided by the authors with concrete comparisons of regional versus global studies. Here, it would be useful if the literature referred to could be expanded. For example, a critical discussion with findings of higher opportunity costs like presented by Regelmann et al. or similar studies would increase the value of the study for policy makers. The authors could get more explicit on how global models that lack the specificity of national or regional forest situations are giving better advice to policy makers regarding opportunity costs of conservation. The manuscript is not yet very convincing here. Regelmann C, Rosenkranz L, Seintsch B, Dieter M (2023) Economic evaluation of different implementation variants and categories of the EU Biodiversity Strategy 2030 using forestry in Germany as a case study. Forests 14(6):1173, DOI:10.3390/f14061173

Response to general comment 5*:* Thank you for this feedback. We would like to clarify that we didn't mean to indicate that regional studies are "less valuable," but rather that they answer different questions. Regional models are essential for deciding when and how to act on local conservation policies, while global models are particularly useful for evaluating system-wide opportunity costs, leakage, and distributional effects across countries. In the case of our study, the global modeling approach uncovers the net impacts of concerted, multilateral action. We also note that a promising direction for future work is a hybrid modeling approach that integrates global market dynamics with region-specific ecological and management constraints. We have expanded the discussion to address this point as follows:

"The substantial differences we observe between global results and country-level outcomes highlight the importance of distinguishing between regional analyses and global market-integrated assessments when evaluating the opportunity costs of large-scale conservation commitments such as 30×30. Regional studies provide essential detail on local forest conditions, management practices, and ecological constraints. For example, Regelmann et al.[29] show that implementing the EU Biodiversity Strategy in Germany could reduce harvest volumes by 13–44% and lower forest-owner revenues by 14–45%, driven in part by increased deadwood retention requirements that divert 15–19% of merchantable wood from the market. Such studies are indispensable for designing national implementation strategies and understanding local trade-offs. However, regional models cannot capture the system-wide market adjustments that occur when conservation policies are implemented across multiple countries. Reductions in timber supply in one region may raise global prices, shift production to other countries, or alter international trade flows, mechanisms that directly influence opportunity costs but lie outside the scope of national or sub-national analyses. As a result, regional studies may overstate opportunity costs when interpreted

in a global policy context, because they do not account for compensatory production or scarcity-driven price effects elsewhere.

Our global modeling approach complements this literature by capturing these cross-border dynamics and provides estimates of system-wide producer opportunity costs under coordinated international conservation efforts. While global models necessarily abstract from local ecological and institutional detail, they offer critical insights into leakage, redistribution of production, and the distributional consequences of conservation across countries. Taken together, these perspectives underscore the value of a hybrid approach, one that integrates global market dynamics with region-specific ecological and management constraints, to support equitable and effective policy design for achieving 30×30."

General comment 6: An important aspect of the costs of conservation are avoided costs of damage to ecosystems. Avoided costs or the value of the ecosystem services that would have been lost if an area was not protected are as relevant as opportunity costs of the protection. The manuscript is not making reference to avoided costs. I think it is necessary to put both concepts into relation. I assume that avoided costs would be an additional item in the cost-benefit analysis. How would this change the results? I understand that this requires additional data different from what the authors have collected. However, there should be at least some brief discussion on the approach and how it contributes to more appropriate estimates of cost-benefit analyses of forest conservation.

Response to general comment 6: Thank you for your feedback. We agree that avoided damages and the value of ecosystem services preserved through conservation are essential components of a full social cost–benefit analysis. Our study focuses specifically on producer opportunity costs within the forest sector, and we now clarify more explicitly that this represents only one part of the total economic cost. The following text was added in the revised manuscript to address this comment:

"A further limitation of this analysis is that it does not capture the full set of societal or economic costs associated with expanding protected forest areas such as avoided damage values or impacts on final consumers. We did not quantify these values here, because our interest was in the producer opportunity cost, which drives most of the perceived cost of protecting global forests, most of the powerful political opposition to expanding land conservation, and which (by definition) was the commercial value of the foregone wood products. Nevertheless, we note that there are possible all-of-society impacts from forest protection that a forestry model cannot capture on its own. On the negative side, the benefit to producers of receiving higher prices implies a disbenefit to the consumers paying those prices, although the distribution of that change across

potentially billions of wood-product consumers has very complex equity and economic implications, lying beyond the scope of the model here. On the positive side, a comprehensive social cost–benefit assessment would also require valuing the economic and societal losses avoided thanks to forest protection, such as biodiversity losses, diminished water regulation, flood mitigation, soil retention, carbon storage, and other regulating and cultural services. Existing evidence emphasizes the magnitude of these benefits: for example, Kappen et al.[30] estimate that the non-commercial social, environmental, and climate-regulatory value of global forests constitutes more than 90% of total forest value, with lower and upper bounds of 50–150 trillion dollars. Such avoided-damage values would generally reduce net social costs of forest conservation and may even yield net economic benefits, in ways that further interact with the producer and consumer effects associated directly with wood products."

General Comment 7: The authors mention that it is likely that there will be substitution of wood products by other material (line 129). However, they do not state at all whether they consider such effects, what difference it would make and how such effects could be included.

Response to general comment 7: Thank you for your feedback. We acknowledge that substitution between wood products and alternative materials (e.g., steel, concrete, plastics) can significantly influence the overall economic and environmental outcomes of forest conservation policies. As noted, our analysis uses a partial equilibrium model focused on the forest sector, and by design, it does not capture cross-sectoral interactions or substitution effects outside the forest products market.

If substitution were considered, reduced wood availability could lead to increased demand for alternative materials, potentially raising costs in other sectors and shifting the overall cost burden. Similarly, if wood (a relatively low-carbon material) is replaced by steel or concrete, substitution could result in higher life-cycle emissions, diminishing the climate benefits of forest conservation.

To incorporate substitution effects, a multi-sectoral modeling approach would be required, such as: Computable General Equilibrium (CGE) models or Integrated Assessment Models (IAMs) that link forest sector dynamics with other sectors in the economy.

We added following text in the revised manuscript addressing this comment:

"This analysis relies on a partial market equilibrium modelling framework that focuses exclusively on the forestry sector. By design, the model holds conditions in other sectors constant and therefore does not capture cross-sectoral shifts that may arise when conservation policies alter land availability or timber supply. Potential interactions with

agriculture, energy, and other land-using sectors, such as shifts in crop production, changes in biomass demand, or broader land-use competition, therefore remain outside the scope of this analysis and warrant further investigation. In addition, partial market equilibrium approaches cannot represent macroeconomic feedbacks or financial-sector channels such as shifts in land values, collateral dynamics, or credit conditions (e.g., see Kedward and Poupard[15]). The results reported here therefore should be interpreted as sector-specific effects rather than comprehensive economy-wide outcomes.

Similarly, partial market equilibrium models such as GFPM cannot capture how reduced timber availability or higher wood prices might affect non-wood material-using industries (e.g., steel, concrete, engineered composites), nor do they show how feedbacks from those sectors might in turn influence wood product markets. The absence of explicit substitution dynamics has two important implications. First, if higher timber prices induce greater use of alternative materials, the resulting reduction in wood demand could moderate some of the price and revenue effects estimated here. Second, substitution may generate environmental spillovers that fall outside the scope of a forestry sector model. For example, replacing wood (a comparatively low carbon construction material) with steel or concrete could increase life cycle emissions, thereby offsetting some of the climate benefits associated with forest conservation. Conversely, in applications where substitution is technologically or economically constrained (e.g., see Kedward and Poupard[15]), the effects estimated by the model may be closer to realized outcomes. Because these dynamics are not represented, the estimates reported here should be interpreted as upper bound estimates under the assumption of limited substitutability across material classes. Incorporating substitution effects would require a multi sectoral modelling framework, such as computable general equilibrium (CGE) models or integrated assessment models (IAMs). However, while CGE models can represent cross sectoral interactions, such models typically sacrifice the detailed representation of forest product markets that a partial-market equilibrium model provides. The present analysis therefore prioritizes sectoral detail over economy wide integration, and the findings should be interpreted within the boundaries of a forestry sector."

Some minor errors or lack of clarity was noticed at the following points of the manuscript:
Specific comment 1. Line 7: term "30x30" should be explained in the abstract

Response to specific comment 1: We added the clarifying text in the abstract to explain the 30x30 as follows*: "… a global effort to designate 30% of Earth's land and ocean area as protected by 2030 under the Convention of Biodiversity (Target 3)."*

Specific comment 2. Line 59: Seems there is a second verb missing in the sentence starting “We explain…”.

Response to specific comment 2*:* We added the phrase ‘identify’ to make the original text clearer as follows:

“We explain why this occurs and identify countries most affected”.

Specific comment 3. Line 72: GFPM is mentioned here for the first time and needs to be introduced

Response to specific comment 3*:* Done

Specific comment 4. Lines 124 and 136: I find the use of the unit “per country” not very useful. What is the reason?

Response to specific comment 4*:* In the original manuscript, this unit was used to express average annual values across all 180 countries represented in the model. Our intention was to communicate that the reported figures represent the mean effect per country (global sum of cumulative NOV divided by 180 countries), similar to per capita GDP measure.

Specific comment 5. Lines 257-259: Repetition of first lines of paragraph above “The GFPM generates…”

Response: Thank you for catching this error. This has been corrected.

Specific comment 6. Fig1-3: Provide an explanation of the scenario abbreviation in figure captions that describe the character of the scenario

Response: Done. See the first response to General comment 4.

Specific comment 7. Tab S5-S7: Please revise table captions. It is not clear to the reader what Tab S6 presents in difference to Tab S5. Make the table captions fully consistent that from one glance the differences between the tables are clear.

Response: Thank you for your suggestion. Table 6 shows the projected changes in NOV under the base (original) price elasticity. Table 5 and 7 show the same outcomes using higher and lower price elasticities. Table 6 caption was edited to clarify this as follows*:*

"Estimated changes in Net Output Values (billion 2018 US $, cumulative, 2025-2060) in world regions in 30x30 scenarios relative to reference scenario, using base price elasticity. Top rows: NOVs with mean cost; bottom rows: NOVs with 5% and 10% higher and lower costs than mean harvest costs."

Response to Reviewer #2's comments

****

General comment 1: The angle of the manuscript is refreshing. It is intuitive but has been overlooked in previous studies. I find the methodology to be solid and the analysis, in general, rigorous. However, I think the framing of the results is problematic and needs further work.

My biggest concern is how "economic cost" is defined in the manuscript. The analysis focuses on net output value (NOV) for producers rather than on economic welfare more broadly. While NOV is a valid metric, interpreting increases in NOV as evidence of low economic costs is potentially misleading. To assess the big picture, impacts on the consumer side (e.g., welfare losses due to price increases and downstream effects) needs to be addressed more explicitly.

Response to general comment 1: Thank you for your feedback. We appreciate the reviewer's concern regarding the interpretation of "economic cost" in the manuscript. In the revised manuscript, we further clarified that our study does not capture the full set of societal or economic costs associated with expanding protected forest areas such as avoided damage values or impacts on final consumers. We added the following clarifying text in the main text and in the supplementary material:

Main text:

"A further limitation of this analysis is that it does not capture the full set of societal or economic costs associated with expanding protected forest areas such as avoided damage values or impacts on final consumers. We did not quantify these values here, because our interest was in the producer opportunity cost, which drives most of the perceived cost of protecting global forests, most of the powerful political opposition to expanding land conservation, and which (by definition) was the commercial value of the foregone wood products. Nevertheless, we note that there are possible all-of-society impacts from forest protection that a forestry model cannot capture on its own. On the negative side, the benefit to producers of receiving higher prices implies a disbenefit to the consumers paying those prices, although the distribution of that change across potentially billions of wood-product consumers has very complex equity and economic implications, lying beyond the scope of the model here. On the positive side, a comprehensive social cost–benefit assessment would also require valuing the economic and societal losses avoided thanks to forest protection, such as biodiversity losses,

diminished water regulation, flood mitigation, soil retention, carbon storage, and other regulating and cultural services. Existing evidence emphasizes the magnitude of these benefits: for example, Kappen et al.[30] estimate that the non-commercial social, environmental, and climate-regulatory value of global forests constitutes more than 90% of total forest value, with lower and upper bounds of 50–150 trillion dollars. Such avoided-damage values would generally reduce net social costs of forest conservation and may even yield net economic benefits, in ways that further interact with the producer and consumer effects associated directly with wood products."

Supplementary material:

"We quantified the economic effects of 30x30 on forest product producers, finding that constrained supply would cause them to be paid more per unit and thus, counterbalance (or even overcompensate) any volume losses from increased land conservation. An increase in forest product prices implies negative effects on forest product consumers, in further counterbalance to the zero-sum or positive effects found for forest product producers. Although these welfare effects are important, our modelling focused on the opportunity costs to producers, for both a conceptual and practical reason. Conceptually, the "cost" of forest protection, especially in the arena of climate change policy, refers specifically to the opportunity cost to producers and not to broader welfare. Since the main drive of the article is that those costs have been exaggerated, we preserve the same sense of cost as "producer opportunity cost". Moreover, the politically meaningful opposition to land conservation will typically come from producers fearing an opportunity cost, and not from consumers. On the pragmatic side, GFPM is also specifically designed to estimate producer effects rather than society-wide welfare effects.

We therefore emphasize that our analysis focuses on changes in NOV for roundwood producers, not the changes in producer and consumer economic surplus across the entire wood products sector. GFPM and comparable forest-sector models do not represent consumer or producer behavior for all products using explicit demand and supply equations, preventing estimates of total welfare. In GFPM, roundwood consumption is derived from the consumption of finished products (sawnwood, panels, and paper) and intermediate products (wood pulp) using input–output coefficients, rather than from a demand curve with an associated elasticity, preventing estimates of roundwood consumer surplus. Likewise, production of finished wood products is not determined by a supply curve but is derived from input–output relationships, which prevents estimation of producer surplus for downstream industries. As a result, the broader effects on roundwood consumers, downstream producers and consumers of finished products, and overall social welfare remain unknown and warrant future investigation using modelling frameworks that include explicit demand and supply functions for all types of timber and finished wood products."

General comment 2: A related concern is that substitution effects are treated rather lightly. While substitution away from wood products is mentioned in the discussion, it is not explored to the extent warranted, given the long time horizon and the important role of price effects in driving the changes.

Response to general comment 2: Thank you for your comment. As noted in the original manuscript, we acknowledged that material substitution—both away from wood products when timber prices rise and toward wood when alternative materials become more expensive—can influence market dynamics and welfare outcomes. However, by design, a partial-equilibrium model holds conditions in all non-forest sectors constant. As a result, the GFPM model cannot capture how reduced timber availability or higher wood prices might affect non-wood material-using industries, nor can it track the feedbacks from those sectors to the forestry sector. This structural limitation is now highlighted in the revised manuscript, and we provided the discussion of how our results would have been affected due to potential inter-sectoral substitution as follows:

"Although our study offers important insights on potential forest-sector responses of expanded forest protection under 30x30, several inherent limitations remain, and therefore findings should be interpreted with caution. This analysis relies on a partial market equilibrium modelling framework that focuses exclusively on the forestry sector. By design, the model holds conditions in other sectors constant and therefore does not capture cross-sectoral shifts that may arise when conservation policies alter land availability or timber supply. Potential interactions with agriculture, energy, and other land-using sectors, such as shifts in crop production, changes in biomass demand, or broader land-use competition, therefore remain outside the scope of this analysis and warrant further investigation. In addition, partial market equilibrium approaches cannot represent macroeconomic feedbacks or financial-sector channels such as shifts in land values, collateral dynamics, or credit conditions (e.g., see Kedward and Poupard[15]). The results reported here therefore should be interpreted as sector-specific effects rather than comprehensive economy-wide outcomes.

Similarly, partial market equilibrium models such as GFPM cannot capture how reduced timber availability or higher wood prices might affect non-wood material-using industries (e.g., steel, concrete, engineered composites), nor do they show how feedbacks from those sectors might in turn influence wood product markets. The absence of explicit substitution dynamics has two important implications. First, if higher timber prices induce greater use of alternative materials, the resulting reduction in wood demand could moderate some of the price and revenue effects estimated here. Second, substitution may generate environmental spillovers that fall outside the scope of a forestry sector model. For example, replacing wood (a comparatively low carbon construction material) with steel or concrete could increase life cycle emissions, thereby

offsetting some of the climate benefits associated with forest conservation. Conversely, in applications where substitution is technologically or economically constrained (e.g., see Kedward and Poupard[15]), the effects estimated by the model may be closer to realized outcomes. Because these dynamics are not represented, the estimates reported here should be interpreted as upper bound estimates under the assumption of limited substitutability across material classes. Incorporating substitution effects would require a multi sectoral modelling framework, such as computable general equilibrium (CGE) models or integrated assessment models (IAMs). However, while CGE models can represent cross sectoral interactions, such models typically sacrifice the detailed representation of forest product markets that a partial-market equilibrium model provides. The present analysis therefore prioritizes sectoral detail over economy wide integration, and the findings should be interpreted within the boundaries of a forestry sector."

General comment 3: The results show substantial distributional heterogeneity across countries, but this is largely glossed over in the discussion. Given the policy relevance of 30x30, these distributional effects deserve more attention, particularly if the authors are aiming for global policy-relevance.

Response to general comment 3: Thank you for your feedback. To address this concern, we have expanded the discussion to more clearly articulate the policy implications of this uneven distribution of impacts. Specifically, we now highlight that countries with large export-oriented forest sectors tend to experience stronger NOV gains due to scarcity-driven price increases, whereas countries with smaller or more domestically oriented sectors may see more modest effects. We also note that these differences underscore the need for targeted international support, especially for countries that may face adjustment challenges or have limited capacity to capture the benefits of higher global prices.

The following text was added in the revised manuscript reflecting those points:

"Our analysis finds that the magnitude and direction of NOV effects vary widely, reflecting differences in baseline production structures, trade orientation, processing capacity, and the relative importance of forest-based industries. Countries with large, export-oriented forest sectors tend to experience stronger NOV gains driven by scarcity-induced price increases, whereas countries with smaller or more domestically oriented sectors may see modest gains or NOV losses. These patterns are consistent with broader evidence that the economic consequences of large-scale forest protection are not uniform across regions and such distributional heterogeneity across countries has important implications for the global implementation of the 30×30 target.

Such heterogeneity suggests the need for policy designs that explicitly account for uneven impacts across countries. Nations that benefit less from scarcity-driven price effects—or that face higher adjustment costs—may require targeted international support, including financial transfers, capacity-building, or mechanisms to help them capture a greater share of value added in forest-product markets. Conversely, countries that experience larger NOV gains may be better positioned to contribute to global conservation financing. Recognizing these distributional patterns is essential for developing equitable and politically feasible pathways for achieving 30×30, also to avoid unequal distributions of protected area expansion. Future work should explore these cross-country differences more deeply, including how they interact with broader welfare effects, substitution dynamics, and the institutional capacity required to implement large-scale forest protection."

General comment 4: To summarize, I think the results are noteworthy, and the analysis fills an important gap in the literature. The methodology itself is standard for the forest economics field and sound (no original contribution, though), but the interpretation and conclusions would benefit from more careful framing and stronger alignment with what the analysis actually demonstrates. The work appears reproducible. Once again, the claims would be more convincing with a clearer distinction between producer outcomes and broader economic impacts/welfare.

Response to general comment 4: Thank you for your thoughtful feedback, which has substantially improved our manuscript. We have addressed all the concerns you raised to the fullest extent possible, as detailed in our responses to each individual comment above.

Response to Reviewer #3's comments

****

General comment 1: It is true that some studies have indicated that there could be high opportunity costs associated with conserving forest ecosystems at very large scales (primarily in the context of conserving them as carbon sinks). However, the authors overstate the case that analyses with models that account for economic feedbacks have not been undertaken, and that (without any nuance in the abstract) "protecting earth's remaining forests...is thought to represent trillions of dollars of lost income". Several recent studies (albeit some are "grey literature") have concluded that the opportunity costs of implementing the 30 x 30 are quite low, or could even provide net benefits (see the very nice synthesis in Kedward & Poupard 2024). This includes the study (Waldron et al., citation 16) on which the forestry scenarios in this manuscript are based. It is only in the discussion that this and other work reaching similar conclusions is provided for context. The work in this manuscript is very important, but it isn't the first

to come to similar conclusions and this should be made clear in the title, abstract and introduction.

Response to comment 1: Thank you for your feedback. We agree that a few recent studies have concluded that the opportunity costs of meeting the 30×30 targets may be relatively low, or in some cases even yield net economic benefits.

The Waldron et al. (2020) study, whose scenarios we've adopted here, finds that large-scale forest conservation can generate positive economic outcomes. However, their estimates are based on gross output changes in the forestry sector and do not deduct harvest and transport costs. As a result, their results cannot be interpreted as net output values.

We appreciate the reviewer's reference to Kedward & Poupard (2024). Their synthesis highlights that a few have explored the potential effects of 30x30 and they find that the economic costs of forest protection policies are often modest, and in some cases net positive, particularly when accounting for the value of ecosystem services, reduced climate related damages, and the stabilizing effects of conservation on long term economic systems.

We have revised the introduction to reflect more clearly that our contribution builds on, rather than precedes, this emerging body of work as follows:

"

We note here that a few recent studies have found similar outcomes, i.e., these costs may be far lower than previously assumed, and in some cases even net beneficial once broader economic feedbacks, avoided damages, and ecosystem-service benefits are considered. For example, Kedward and Poupard[15] synthesize recent modelling exercises and report that the opportunity costs of implementing the 30×30 target are often modest, particularly when accounting for the value of ecosystem services, reduced climate related damages, and the stabilizing effects of conservation on long term economic systems. Similarly, Waldron et al.[16], whose scenarios underpin the conservation pathways examined here, report positive economic effects from expanding protected areas. However, their estimates reflect gross output changes in the forestry sector and do not deduct harvest and transport costs, making them unsuitable for assessing forestry sector net output values. Similarly, following Waldron et al.'s[16] approach, Johnson et al.[17] estimated the cost of implementing 30x30 policies using an integrated ecosystem-economy model (GTAP-Invest) and found the total cost of such policies to be modest at $115 billion (0.1% of global GDP), and to be only $13 billion if the economic benefits of avoided CO_2 emissions were accounted for.

The present study builds on this literature by providing a net output perspective that explicitly incorporates production costs within a sectorally detailed modelling framework

that captures projected shifts in prices, production, consumption and trade of roundwood because of increased forest protection. By focusing on net output value within the forestry sector, our analysis complements existing work and helps refine the understanding of how opportunity costs behave under large-scale forest protection scenarios"

We've also added following text in the abstract acknowledging that the recent studies support our findings as follows:

"Emerging studies support our findings that cost of forest conservation may be far lower than previously assumed, and in some cases even net beneficial once broader economic feedbacks, avoided damages, and ecosystem service benefits are considered."

General comment 2: Opportunity costs are not the only costs associated with massively increasing global protected areas. Other costs include, but are not limited to administration, enforcement, protected-area infrastructure, monitoring, etc. This analysis was not designed to account for these costs, but these costs could be very high and should be acknowledged. (Note that the title is currently "Reports of the economic cost of global forest protection have been greatly exaggerated". This manuscript does not account for total economic cost).

Response to general comment 3: Thank you for your suggestion. We agree that a full accounting of the "total economic cost" of large-scale forest protection would also need to include a range of implementation and management costs, such as administration, enforcement, monitoring, protected-area infrastructure, and community engagement. These costs can be substantial, particularly in regions with limited institutional capacity, and our analysis was not designed to quantify them. We also note that avoided damages and the value of ecosystem services preserved through conservation are essential components of a full social cost–benefit analysis, which was outside the scope of our analysis. We now explicitly acknowledge these limitations in the revised manuscript.

The following text was added in the revised manuscript to address this comment:

"A further limitation of this analysis is that it does not capture the full set of societal or economic costs associated with expanding protected forest areas such as avoided damage values or impacts on final consumers. We did not quantify these values here, because our interest was in the producer opportunity cost, which drives most of the perceived cost of protecting global forests, most of the powerful political opposition to expanding land conservation, and which (by definition) was the commercial value of the foregone wood products. Nevertheless, we note that there are possible all-of-society impacts from forest protection that a forestry model cannot capture on its own. On the

negative side, the benefit to producers of receiving higher prices implies a disbenefit to the consumers paying those prices, although the distribution of that change across potentially billions of wood-product consumers has very complex equity and economic implications, lying beyond the scope of the model here. On the positive side, a comprehensive social cost–benefit assessment would also require valuing the economic and societal losses avoided thanks to forest protection, such as biodiversity losses, diminished water regulation, flood mitigation, soil retention, carbon storage, and other regulating and cultural services."

General comment 3: The authors need to justify the sensitivity analysis for price elasticity. They state that "we adjusted the price elasticity parameter in either direction by +/- 15%". Why this range? This seems to be a relatively narrow range for a sensitivity analysis (see comments below on product substitution). Where are the references/justification for this? Increasing price elasticity by much more that 15% could well result in a scenario where opportunity costs are high. Why wasn't this possibility explored? Without a strong justification for the range of elasticity explored in the manuscript, I would think it essential to extend the analysis to a much wider range of elasticities.

Response to general comment 3: Thank you for your suggestion. Our choice of a ±15% adjustment to the price elasticity parameter was motivated by the specific purpose of this test: to identify whether there exists a plausible elasticity range within which the net output value (NOV) would turn negative, given the structure of the Global Forest Products Model (GFPM). We particularly note that the price sensitivity parameter interacts with the cost sensitivity parameter, where the cost parameter has by far the larger effect size. In other words, a large additional change in the price elasticity would be captured by a relatively small change in the assumed cost. We therefore felt that, since our cost parameter bracketed a range that seemed at the limit of commercial likelihood, then the overall sensitivity of our conclusions to the combined effects of cost uncertainty and price uncertainty had been reasonably covered.

General comment 4: The authors should do a better job of highlighting the limitations of an analysis that uses a partial-equilibrium model that focuses exclusively on the forestry sector. For example, the analysis does not account for spillover effects on other sectors and markets such as agriculture and energy. As another example, this analysis doesn't account for material substitution. For example, if prices for forestry products rise substantially (which is an outcome of this analysis), consumers may find substitutes thereby reducing demand. This has been taken into account to some extent by examining elasticity, but this does not explicitly account for product substitution which may or may not be easily reversible. There are several other major limitations of a

partial-equilibrium model that focuses exclusively on the forestry sector that mean that much greater caution is necessary when interpreting the results of this analysis. Overall, the discussion should be much more open about the limitations associated with the methods and assumptions.

Response to general comment 4: Thank you for your suggestion. We agree that the inherent limitations of partial equilibrium model cannot capture the full set of economy-wide adjustments that may occur when conservation policies alter land availability, timber supply, or forest management practices. These interactions may either amplify or dampen the effects we estimate. We now highlight this point more clearly in the discussion.

We also acknowledge that a partial market equilibrium model such as GFPM cannot not fully capture the effects of potential material substitution. If rising timber prices induce consumers or firms to switch toward alternative materials, such as steel, concrete, or engineered composites, the resulting reduction in timber demand could mitigate some of the price and NOV effects we report. Because our model does not explicitly represent substitution across material classes, we now emphasize that our results should be interpreted as upper-bound estimates of price and surplus impacts under the assumption of limited substitution.

Our goal is to offer a transparent, tractable examination of the direct impacts within the forestry sector, which remains a critical component of the broader land-use system, rather than provide a full general-equilibrium assessment. We now make this positioning explicit and clarify that our findings should be viewed as one input into a larger, multi-sectoral evidence base.

We added several paragraphs in the main text and supplementary material highlighting those points as follows:

Main text:

Although our study offers important insights on potential forest-sector responses of expanded forest protection under 30x30, several inherent limitations remain, and therefore findings should be interpreted with caution. This analysis relies on a partial market equilibrium modelling framework that focuses exclusively on the forestry sector. By design, the model holds conditions in other sectors constant and therefore does not capture cross-sectoral shifts that may arise when conservation policies alter land availability or timber supply. Potential interactions with agriculture, energy, and other land-using sectors, such as shifts in crop production, changes in biomass demand, or broader land-use competition, therefore remain outside the scope of this analysis and warrant further investigation. In addition, partial market equilibrium approaches cannot represent macroeconomic feedbacks or financial-sector channels such as shifts in land

values, collateral dynamics, or credit conditions (e.g., see Kedward and Poupard[15]). The results reported here therefore should be interpreted as sector-specific effects rather than comprehensive economy-wide outcomes.

Similarly, partial market equilibrium models such as GFPM cannot capture how reduced timber availability or higher wood prices might affect non-wood material-using industries (e.g., steel, concrete, engineered composites), nor do they show how feedbacks from those sectors might in turn influence wood product markets. The absence of explicit substitution dynamics has two important implications. First, if higher timber prices induce greater use of alternative materials, the resulting reduction in wood demand could moderate some of the price and revenue effects estimated here. Second, substitution may generate environmental spillovers that fall outside the scope of a forestry sector model. For example, replacing wood (a comparatively low carbon construction material) with steel or concrete could increase life cycle emissions, thereby offsetting some of the climate benefits associated with forest conservation. Conversely, in applications where substitution is technologically or economically constrained (e.g., see Kedward and Poupard[15]), the effects estimated by the model may be closer to realized outcomes. Because these dynamics are not represented, the estimates reported here should be interpreted as upper bound estimates under the assumption of limited substitutability across material classes. Incorporating substitution effects would require a multi sectoral modelling framework, such as computable general equilibrium (CGE) models or integrated assessment models (IAMs). However, while CGE models can represent cross sectoral interactions, such models typically sacrifice the detailed representation of forest product markets that a partial-market equilibrium model provides. The present analysis therefore prioritizes sectoral detail over economy wide integration, and the findings should be interpreted within the boundaries of a forestry sector.

A further limitation of this analysis is that it does not capture the full set of societal or economic costs associated with expanding protected forest areas such as avoided damage values or impacts on final consumers. We did not quantify these values here, because our interest was in the producer opportunity cost, which drives most of the perceived cost of protecting global forests, most of the powerful political opposition to expanding land conservation, and which (by definition) was the commercial value of the foregone wood products. Nevertheless, we note that there are possible all-of-society impacts from forest protection that a forestry model cannot capture on its own. On the negative side, the benefit to producers of receiving higher prices implies a disbenefit to the consumers paying those prices, although the distribution of that change across potentially billions of wood-product consumers has very complex equity and economic implications, lying beyond the scope of the model here. On the positive side, a comprehensive social cost–benefit assessment would also require valuing the economic

and societal losses avoided thanks to forest protection, such as biodiversity losses, diminished water regulation, flood mitigation, soil retention, carbon storage, and other regulating and cultural services. Existing evidence emphasizes the magnitude of these benefits: for example, Kappen et al.30 estimate that the non-commercial social, environmental, and climate-regulatory value of global forests constitutes more than 90% of total forest value, with lower and upper bounds of 50–150 trillion dollars. Such avoided-damage values would generally reduce net social costs of forest conservation and may even yield net economic benefits, in ways that further interact with the producer and consumer effects associated directly with wood products."

Supplementary material:

"We quantified the economic effects of 30x30 on forest product producers, finding that constrained supply would cause them to be paid more per unit and thus, counterbalance (or even overcompensate) any volume losses from increased land conservation. An increase in forest product prices implies negative effects on forest product consumers, in further counterbalance to the zero-sum or positive effects found for forest product producers. Although these welfare effects are important, our modelling focused on the opportunity costs to producers, for both a conceptual and practical reason. Conceptually, the "cost" of forest protection, especially in the arena of climate change policy, refers specifically to the opportunity cost to producers and not to broader welfare. Since the main drive of the article is that those costs have been exaggerated, we preserve the same sense of cost as "producer opportunity cost". Moreover, the politically meaningful opposition to land conservation will typically come from producers fearing an opportunity cost, and not from consumers. On the pragmatic side, GFPM is also specifically designed to estimate producer effects rather than society-wide welfare effects.

We therefore emphasize that our analysis focuses on changes in NOV for roundwood producers, not the changes in producer and consumer economic surplus across the entire wood products sector. GFPM and comparable forest-sector models do not represent consumer or producer behavior for all products using explicit demand and supply equations, preventing estimates of total welfare. In GFPM, roundwood consumption is derived from the consumption of finished products (sawnwood, panels, and paper) and intermediate products (wood pulp) using input–output coefficients, rather than from a demand curve with an associated elasticity, preventing estimates of roundwood consumer surplus. Likewise, production of finished wood products is not determined by a supply curve but is derived from input–output relationships, which prevents estimation of producer surplus for downstream industries. As a result, the broader effects on roundwood consumers, downstream producers and consumers of finished products, and overall social welfare remain unknown and warrant future

investigation using modelling frameworks that include explicit demand and supply functions for all types of timber and finished wood products."

References:

Johnson *et al.* 2021.*The Economic case for nature: A global Earth-Economy Model to assess development policy pathways*. https://www.worldbank.org/en/topic/environment/publication/the-economic-case-for-nature.

Kappen G, Kastner E, Kurth T, Puetz J, Reinhardt A, Soininen J. The Staggering Value of Forests—and How to Save Them. Boston Consulting Group; 2020. https://www.bcg.com/publications/2020/the-staggering-value-of-forests-and-how-to-save-them.

Kedward & Poupard. 2024. *The economic and financial risks of implementing the '30x30' Global Biodiversity Framework Targets*. https://www.ucl.ac.uk/bartlett/public-purpose/wp2024-13.

Regelmann, C., Rosenkranz, L., Seintsch, B. & Dieter, M. Economic evaluation of different implementation variants and categories of the EU Biodiversity Strategy 2030 using forestry in Germany as a case study. Forests 14, 1173 (2023).

Waldron, A. *et al.* 2020. Protecting 30% of the planet for nature: costs, benefits and economic implications. https://www.conservation.cam.ac.uk/files/waldron_report_30_by_30_publish.pdf.
